# Biomaterials targeting senescent cells for bone regeneration: State-of-the-art and future perspectives

**DOI:** 10.1016/j.bioactmat.2025.09.002

**Published:** 2025-09-08

**Authors:** Haitong Wu, Qing Zhang, Jinhao Zhu, Lihong Wu, Yin Xiao, Xuechao Yang

**Affiliations:** a Department of Basic Oral Medicine, School and Hospital of Stomatology, Guangdong Engineering Research Center of Oral Restoration and Reconstruction, Guangzhou Key Laboratory of Basic and Applied Research of Oral Regenerative Medicine, Guangzhou Medical University, Guangzhou, 510182, China; b School of Medicine and Dentistry & Institute for Biomedicine and Glycomics, Griffith University, Gold Coast, QLD, 4222, Australia; c The Australia-China Centre for Tissue Engineering and Regenerative Medicine (ACCTERM), Brisbane, QLD, 4000, Australia

**Keywords:** Biomaterials, Senescent cells, Senescence-associated secretory phenotype, Bone regeneration, Nanodrug delivery

## Abstract

Bone defect treatment remains a significant clinical challenge, further exacerbated by the demographic transition toward an aging society. In elderly populations, the increased proportion of senescent cells emerges as a fundamental determinant that substantially compromises regenerative outcomes. In senescent bone tissues, the progressive accumulation of senescent cells compromises bone regenerative capacity through multifaceted mechanisms, encompassing both intrinsic functional impairment of senescent cells and the far-reaching impact of the senescence-associated secretory phenotype (SASP) on the surrounding cellular and tissue microenvironment. Advanced biomaterials provide a platform for targeted anti-senescence interventions. One strategy is the selective elimination of senescent cells, achieved by engineering materials as delivery systems for senolytics or as platforms that modulate immune clearance. A more nuanced approach seeks functional rejuvenation, using biomaterials to restore cellular homeostasis by mitigating inflammation, correcting metabolic dysfunction, and reprogramming gene expression. A holistic strategy remodels the senescent microenvironment itself, accomplished through materials designed to restore biochemical homeostasis, provide physical guidance, and reprogram biological communication. This review delineates these material-based strategies, from direct cellular targeting to comprehensive niche remodeling. We also evaluate the significant hurdles to clinical translation, including challenges in biological specificity, preclinical model fidelity, and regulatory pathways. Ultimately, this work provides a conceptual framework for designing next-generation biomaterials to regenerate aging bone tissues.

## Highlights

1. A framework to design biomaterials targeting senescent cells in bone.
2. Biomaterials for senescence: elimination, rejuvenation, microenvironment remodeling.
3. Materials act as smart carriers or have intrinsic seno-modulatory effects.
4. Key translational hurdles are scientific, manufacturing, and regulatory.
5. The field is moving from empirical design to a more predictive science.

## Graphical abstract

**Figure:**
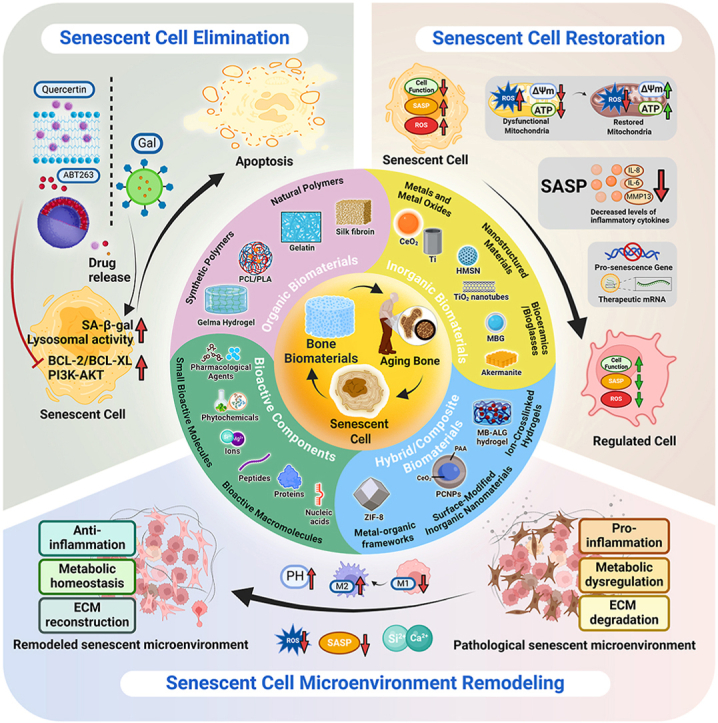

## 1 Introduction

The world is undergoing a profound demographic shift, with the number of persons aged 65 and over projected to surpass the number of children under 18 by 2080 [1]. This demographic trend corresponds with a higher incidence of age-related skeletal disorders, with an estimated 37 million fragility fractures occurring annually worldwide in individuals over the age of 55 [2]. Consequently, the clinical management of bone defects in this geriatric population, whether arising from fragility fractures, trauma, or disease, presents a significant challenge [3]. Despite substantial advancements in bone biomaterials through biomimetic design, microstructural engineering, immunomodulation, and angiogenic promotion, their clinical translation efficacy in geriatric populations remains suboptimal. This phenomenon highlights a critical limitation, namely that conventional biomaterials fail to adequately address the unique cellular and molecular characteristics of the microenvironment inherent to aging bone tissue [[4], [5], [6], [7]].

Aged bone tissue typically manifests as decreased bone mass, compromised mechanical integrity, and significantly impaired regenerative capacity [3,[8], [9], [10], [11], [12], [13]]. These degenerative alterations stem from multiple interconnected pathophysiological mechanisms, including restricted differentiation potential of bone marrow mesenchymal stem cells (BMSCs), dysregulated osteoblast-osteoclast homeostasis, vascular remodeling dysfunction, and perturbed immune microenvironments [[14], [15], [16]]. Among these age-associated bone dysfunctions, the progressive accumulation of senescent cells has been identified as a pivotal regulatory factor. This recognition has prompted a fundamental shift in bone biomaterial engineering. The focus has moved from materials with generalized regenerative functions toward the rational design of platforms that execute specific anti-senescence strategies [4,12,[17], [18], [19], [20]]. These strategies aim to achieve three distinct biological outcomes by selectively eliminating senescent cells, promoting their functional rejuvenation, or remodeling their pathological microenvironment [[21], [22], [23], [24], [25]].

**Fig. 1:**
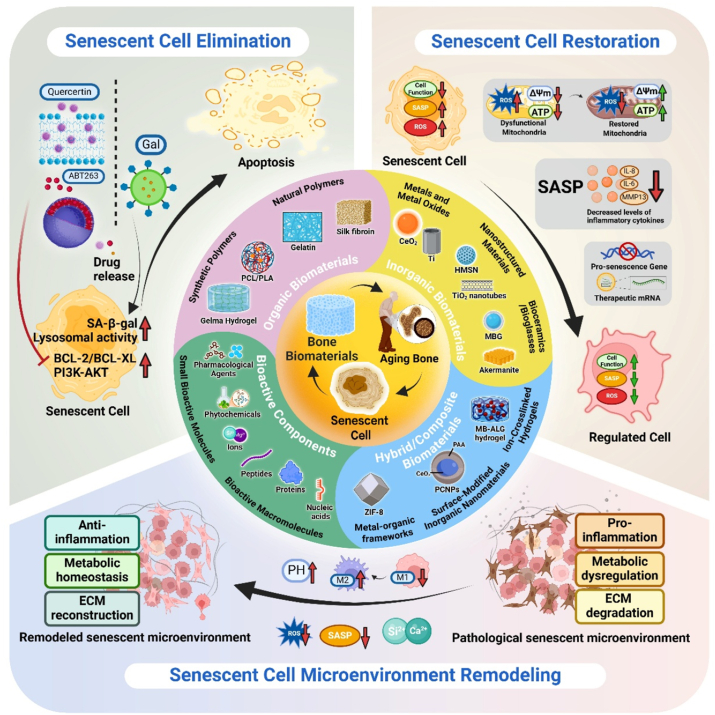
Schematic diagram of senescence-targeting bone biomaterials.

In this comprehensive review, we systematically analyze the emerging design principles of biomaterials targeting senescent cells in aging bone tissue (Fig. 1). We first establish the theoretical foundation by elucidating the molecular characteristics of bone aging and cellular senescence. The core of this review is then structured around the three primary therapeutic strategies of selective elimination, functional rejuvenation, and microenvironment remodeling. Within the discussion of each strategy, we dissect the underlying material-based approaches engineered to achieve these specific biological outcomes, including the design of advanced delivery systems for therapeutics and nucleic acids, the engineering of materials with intrinsic bioactivity, the modulation of physicochemical and mechanical properties, and the development of immuno-modulatory platforms. Through this systematic analysis, we aim to provide an integrative conceptual framework for developing age-specific bone biomaterials while discussing potential future research directions in this rapidly evolving field.

## 2 Senescent cells in bone tissue: molecular characteristics and their impact on regeneration

**Fig. 2:**
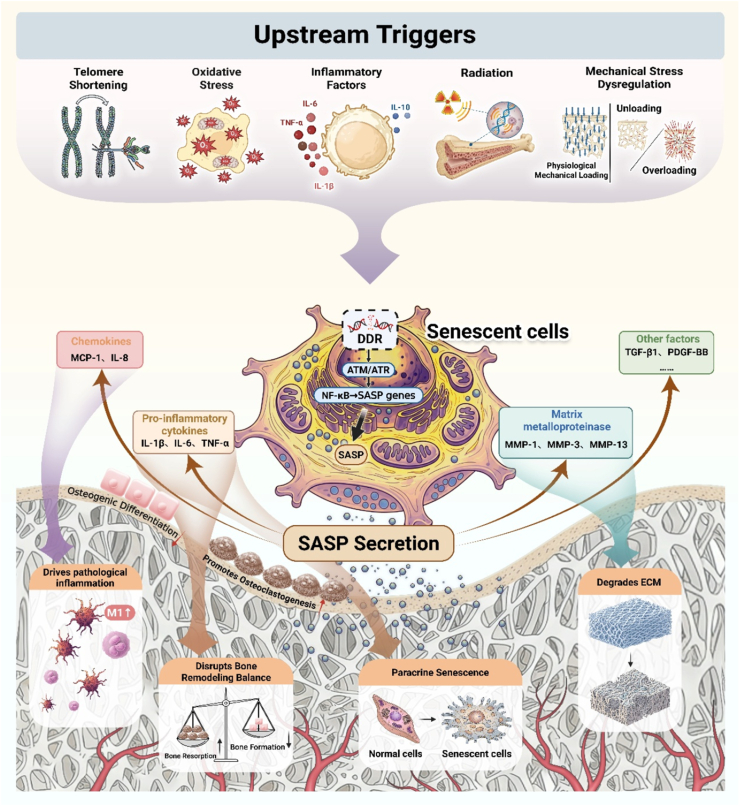
Pathophysiology of cellular senescence in bone tissue. Upstream triggers induce cellular senescence in bone, activating master regulatory pathways such as NF-κB to drive the secretion of the SASP. This secretome then drives bone pathophysiology by promoting pathological immune responses, inducing paracrine senescence, uncoupling bone remodeling towards net resorption, and degrading the extracellular matrix (ECM), ultimately impairing regeneration.

Bone tissue constitutes a dynamically remodeling mineralized structure whose homeostasis is orchestrated through the sophisticated interplay of multiple cell populations [26,27]. With advancing age, bone tissue gradually manifests typical senescent phenotypes, this process involves various cellular components within the bone microenvironment. The bone cells transition into senescent states at different rates and exhibit distinct biochemical characteristics, collectively establishing a complex intercellular signaling network [3,28,29]. Understanding the heterogeneity of cellular senescence in bone tissue and its regulatory mechanisms is crucial for developing targeted therapeutic strategies. Here, we systematically examine the molecular characteristics of senescent cells in bone, their pathophysiological impacts on tissue regeneration, and provide the foundation for subsequent discussions on biomaterial-based intervention approaches. To provide a comprehensive overview of these complex interactions, Fig. 2 illustrates the entire cascade, from the initial triggers of senescence to the establishment of the pathological senescent bone microenvironment, which will be elaborated upon in the following sections.

### 2.1 Biological properties of senescent cells

#### 2.1.1 Molecular signatures and phenotypic features of senescent cells

Senescent cells gradually accumulate throughout various tissues of the organism, particularly with age advancement, exposure to stress, or disease development [30]. Senescent cells exhibit a complex biological phenotype encompassing multiple critical alterations, predominantly characterized by irreversible cell cycle arrest, enhanced anti-apoptotic capacity, and metabolic dysfunction [19,20,31]. This stable cell cycle arrest is regulated by interconnected signaling networks, particularly the p53-Cdkn1a (p21) and RB-Cdkn2a (p16) pathways, the fundamental mechanisms governing cell cycle cessation. The upregulation of p21 and p16 proteins has been established as canonical biomarkers for identifying senescent cells, and in osseous tissue, these pathways also modulate interactions between senescent cells and their surrounding microenvironment, establishing a state of stable growth arrest that must be actively maintained against pro-apoptotic pressures [19,32,33].

The first two of these hallmarks, stable cell cycle arrest and apoptosis resistance, are mechanistically intertwined. The same key cell cycle regulatory pathways that initiate senescence, notably the p53-p21 axis, are also central regulators of apoptosis [34,35]. Consequently, for a cell to persist in a senescent state rather than undergo apoptosis, the active suppression of pro-apoptotic pathways is required. This is mechanistically achieved through the upregulation of anti-apoptotic proteins, with the B-cell lymphoma 2 (BCL-2) family, including BCL-2 and BCL-XL, being critical mediators. This direct linkage between senescence and apoptosis resistance is clearly demonstrated in the bone microenvironment, where senescent mesenchymal cells have been identified by a p16^+^/Ki67^-^/BCL-2^+^ phenotype [36]. This specific marker profile physically co-locates a key senescence marker (p16^+^) and cell cycle exit marker (Ki67^-^) with a principal anti-apoptotic protein (BCL-2^+^) in the same cell population. This dependency on anti-apoptotic pathways drives their accumulation in bone tissue, which in turn disrupts remodeling, contributes to age-related skeletal pathology, and presents a key vulnerability for senolytic therapies to restore regenerative capacity [[37], [38], [39], [40]].

The metabolic alterations in senescent cells manifest through multiple interconnected alterations. A hallmark feature is their enhanced lysosomal activity, particularly evident in increased senescence-associated β-galactosidase (SA-β-gal) expression, which has become a widely adopted marker for senescence identification [41]. This is accompanied by profound mitochondrial changes, characterized by aberrant fission, compromised membrane potential, and elevated reactive oxygen species (ROS) production [[42], [43], [44]]. In bone tissue, these metabolic alterations create a particularly detrimental environment, as increased ROS levels not only maintain the senescent state but also propagate damage to surrounding healthy cells, establishing a self-perpetuating cycle of tissue dysfunction [[45], [46], [47]]. These distinct metabolic signatures not only define the senescent phenotype but also offer promising therapeutic targets, with interventions aimed at restoring mitochondrial function, reducing oxidative stress, or exploiting the heightened lysosomal activity representing potential strategies to counteract senescence-mediated bone pathologies.

#### 2.1.2 The SASP: composition and regulation

Beyond the stable growth arrest, a pivotal functional characteristic of senescent cells is the development of the SASP [20,[48], [49], [50]]. The SASP is a highly complex secretome, broadly composed of pro-inflammatory cytokines, chemokines, growth factors, and matrix-remodeling proteases [51]. More recent evidence has expanded this definition to include bioactive lipids, extracellular vesicles (EVs), and non-coding nucleic acids [[52], [53], [54]]. The precise composition of the SASP is remarkably heterogeneous, varying significantly based on the initial senescence-inducing stimulus and the cell of origin, which underpins its pleiotropic biological effects [55,56].

The production of the SASP is orchestrated by a sophisticated network of intracellular signaling pathways [[56], [57], [58], [59]]. A primary trigger is the persistent DNA damage response (DDR), which initiates a cascade that sustains the secretory program [60]. A parallel and equally critical activation route involves the innate immune cGAS-STING pathway, which senses misplaced cytosolic DNA and robustly induces an inflammatory response [61,62]. These upstream signals often converge on the master transcription factor NF-κB, which directly controls the expression of a vast array of pro-inflammatory SASP genes [[63], [64], [65], [66]]. This core network is further amplified and fine-tuned by other crucial signaling cascades. For example, the p38 MAPK pathway enhances NF-κB transcriptional activity and stabilizes SASP mRNAs, while the mTOR pathway provides another critical layer of regulation by controlling the translation of key SASP components [67,68]. The integration of these core pathways ensures the robust and sustained secretion that enables the potent non-cell-autonomous effects of senescent cells.

### 2.2 Pathophysiological effects of senescent cells on bone tissue

The accumulation of senescent cells profoundly impacts bone tissue homeostasis and regeneration [[69], [70], [71]]. These effects primarily manifest through cell-autonomous dysfunction and non-cell-autonomous signaling pathways. The cell-autonomous consequences arise from the intrinsic functional deterioration of senescent cells, which compromises their physiological roles in bone metabolism, as evidenced by reduced osteoblast mineralization capacity and impaired response to mechanical stimuli [3,72]. Complementing this, the non-cell-autonomous pathway is driven by the cells’ secretory phenotype. This phenotype mediates paracrine effects on neighboring cells and orchestrates broad microenvironmental alterations, such as releasing pro-inflammatory cytokines that shift the bone remodeling balance toward resorption [73]. Together, these mechanisms contribute to the development of age-associated skeletal disorders. To systematically delineate the distinct contributions of each cell type to this pathology, Table 1 provides a comprehensive summary of the senescence-associated characteristics of key cell populations in bone, detailing both their autonomous functional decline and their secretory profiles.

**Table 1:** The cellular basis of senescence in bone regeneration.

Cell type	Primary senescence inducers	Key senescence markers	SASP profile	Functional impact	Molecular mechanisms	Ref.
BMSCs	Replicative exhaustion; Oxidative stress & DNA damage; Inflammatory factors; Glucocorticoids	SA-β-gal+, p16/p21 ↑ Morphology: large/flat; CFU-F ↓; PPARγ ↑	Pro-inflammatory Proteases (MMPs) Pro-senescence miRNAs in Evs	Stemness ↓; Lineage skew: Osteo ↓, Adipo ↑; Bone marrow adipose tissue ↑	Wnt ↓, RUNX2 ↓, PPARγ ↑; Autophagy ↓	[[74], [75], [76], [77], [78]]
Osteoblasts	Oxidative stress; Replicative senescence; DNA damage	SA-β-gal+, p16/p21 ↑ Osteo-markers ↓ (RUNX2); Collagen synth ↓	Pro-osteoclastogenic: RANKL ↑, OPG ↓ Inflammation: IL-6 ↑, MMP-9 ↑	Bone formation ↓; Promotes bone resorption via SASP	p53/p16/RB, mTOR, Wnt/β-catenin, SIRTs, AMPK	[46,79,80]
Osteocytes	Physiological aging; Mechanical unloading; Estrogen deficiency; DNA damage	SA-β-gal+, p16/p21 ↑, γH2AX ↑ Cx43 ↓ Autophagy ↓	Primary SASP source Pro-osteoclastogenic: RANKL ↑, SOST ↑ Pro-inflammatory	Bone loss ↑, Mechanotransduction ↓, peri-lacunar remodeling ↓, lacunar-canalicular network degradation	p53/p16 pathways, GATA4→RANKL ↑, Wnt/TGF-β ↓, JAK-STAT ↑	[3,81,82]
Osteoclasts	Intrinsic (post-mitotic); External (SASP, age); Self-induced (CTSK)	SA-β-gal+, p16/p21 ↑, Hyper-resorptive phenotype	SASP-like (MMP-9 ↑, CTSK ↑); Promotes “inflammaging”	Enhanced bone resorption	Epigenetic dysregulation; Proteostasis imbalance; Mitochondrial dysfunction (ROS ↑); Altered Ca2+ signaling	[15,83]
T-Cells	Persistent antigen stimulation Inflammatory stress (cytokines, ROS) Bacterial factors (LPS, CDT)	SA-β-gal+; Surface: CD28 ↓, CD27 ↓, KLRG1 ↑ Intracellular: DDR ↑, p16 ↑, TCR signaling ↓	Th17-biased SASP Components: RANKL ↑, IL-17A ↑, TNF-α ↑	Drives Th17/Treg imbalance promoting bone resorption; Senescent CD8^+^ inhibit osteoblasts	p38-STAT3 axis activation (via autophagy↓/ROS↑); Metabolic shift towards glycolysis (mTORC1-driven)	[[84], [85], [86]]
Macrophages	Paracrine induction; Chronic inflammation	SA-β-gal+; Impaired pro-inflammatory (M1) phenotype→pro-regenerative (M2) phenotype	Pro-inflammatory (TNF-α); Specific factor: Grancalcin (GCA)	Phagocytosis ↓ → chronic inflammation; GCA inhibits osteogenesis, promotes adipogenesis.	p16/RB, NF-κB↑, GCA/Plexinb2, Autophagy↓ TLR↓	[5,[87], [88], [89]]
Bone marrow adipocytes (BMAds)	Aging, Obesity; Glucocorticoids	SA-β-gal+, p16 ↑; DNA damage ↑ (γH2AX); Nuclear changes (Lamin B1 ↓)	Spreads secondary senescence (Targets: Endothelial cells, OBs); JAKi-sensitive	Impact: Reciprocal to bone mineral density (BMD) ↓;	GC-induced Oxylipin-PPARγ positive feedback loop drives senescence (via INK family)	[90,91]

**Abbreviations**: CFU-F, Colony-forming unit-fibroblast; PPARγ, Peroxisome proliferator-activated receptor gamma; RUNX2, Runt-related transcription factor 2; RANKL, Receptor activator of nuclear factor kappa-B ligand; OPG, Osteoprotegerin; mTOR, mammalian target of rapamycin; SIRTs, Sirtuins; AMPK, AMP-activated protein kinase; γH2AX, gamma H2A histone family member X; Cx43, Connexin 43; SOST, Sclerostin; GATA4, GATA binding protein 4; CTSK, Cathepsin K; CDT, Cytolethal distending toxin; CD28, Cluster of differentiation 28; CD27, Cluster of differentiation 27; KLRG1, Killer cell lectin-like receptor G1; DDR, DNA damage response; TCR, T-cell receptor; mTORC1, mammalian target of rapamycin complex 1; GCA, Grancalcin; BMAds, Bone marrow adipocytes; JAKi, JAK inhibitor; BMD, Bone mineral density; INK family, Inhibitor of cyclin-dependent kinase family.

#### 2.2.1 Cell-autonomous functional decline of bone resident cells

The primary cell-autonomous impact of senescence on bone is the functional decay of resident cells, beginning at the progenitor level with BMSCs [88,92,93]. Senescent BMSCs exhibit diminished self-renewal and a skewed differentiation potential [94,95]. Their capacity for osteogenesis is reduced, linked to the downregulation of transcription factors like RUNX2, while their propensity for adipogenesis is enhanced due to the upregulation of regulators like PPARγ [91,96]. This imbalanced lineage commitment directly leads to a depleted supply of osteoblasts and increased marrow adiposity with age [29].

This functional decay extends to mature osteoblasts. They exhibit a reduced capacity to synthesize and mineralize bone matrix, reflected by lower expression of key osteogenic genes (RUNX2, Osterix) and proteins such as Type I Collagen [97,98]. Moreover, their responsiveness to crucial anabolic signals like BMPs and TGF-β is blunted, further impairing bone formation [99].

Senescence also compromises osteocytes, the primary mechanosensors of bone. Senescent osteocytes display impaired mechanosensitivity, a reduction in dendritic processes, and disrupted perilacunar and canalicular remodeling [81,100]. This functional decline is partly attributed to the reduced expression of Connexin-43 (Cx43), a protein essential for their intercellular communication network [101,102].

This collective cell-autonomous deterioration provides a direct mechanism for age-related bone fragility. However, beyond this passive functional loss, senescent cells also actively corrupt the local microenvironment through deleterious paracrine signaling.

#### 2.2.2 SASP-mediated dysregulation of bone remodeling

In the skeletal context, the SASP acts as the primary driver of non-cell-autonomous pathology by disrupts the balance between bone formation and resorption [103]. Specific components of this complex secretome, such as the pro-inflammatory cytokines TNF-α, IL-6, and IL-1β, concurrently suppress the differentiation and function of osteoprogenitor cells while promoting the formation and activity of osteoclasts [92,[104], [105], [106], [107], [108], [109], [110]]. The inhibition of osteogenesis often involves interference with critical anabolic pathways, such as the JAK/STAT cascade repressing essential osteogenic transcription factors [111,112]. Simultaneously, these inflammatory mediators dysregulate the RANKL/OPG signaling axis, the central determinant of osteoclastogenesis [113]. They stimulate RANKL expression in various local cells, a process compounded by senescent cells themselves becoming significant RANKL sources [114]. This sustained increase in RANKL, often coupled with a reduction in its decoy receptor OPG, shifts the RANKL/OPG ratio to favor a state of persistent, heightened bone resorption [82,115].

This SASP-driven dysregulation is further amplified through bystander senescence, a mechanism of senescence propagation [116]. Factors within the SASP can induce a senescent state in adjacent healthy cells, thereby expanding the population of SASP-producing cells and establishing a self-reinforcing pathological signaling loop [[104], [105], [106], [107]]. However, these catabolic effects specifically reflect chronic SASP activity rather than the transient SASP responses observed during normal wound healing [117]. While acute SASP contributes to physiological repair processes, the persistent secretory profile of established senescent cells drives sustained tissue degradation in aging bone.

#### 2.2.3 Pathophysiology of the senescent bone microenvironment

The convergence of intrinsic cellular decline and persistent SASP-driven signaling establishes a complex, self-perpetuating pathological niche: the Senescent Bone Microenvironment (SBM) [118,119]. This integrated environment represents a critical barrier to bone regeneration in the aging process. The SBM is characterized by a dysfunctional cellular landscape and a compromised acellular milieu that together trap the tissue in a pro-inflammatory and anti-regenerative state [[120], [121], [122], [123]].

The cellular landscape of the SBM comprises a heterogeneous population of compromised cells. It includes bone-resident cells exhibiting functional decay, such as senescent mesenchymal stem cells with skewed adipogenic differentiation and impaired osteogenic potential [124], as well as dysfunctional osteoblasts with reduced matrix production capacity [125]. This population is expanded by SASP-recruited immune cells that adopt a dysfunctional phenotype. Notably, macrophages within this environment predominantly exhibit the M1 pro-inflammatory phenotype rather than the M2 anti-inflammatory and tissue-repairing phenotype [[126], [127], [128], [129]]. Furthermore, senescent endothelial cells contribute to defective angiogenesis, including a reduction in specialized H-type capillaries essential for coupling angiogenesis and osteogenesis, thereby impairing nutrient and oxygen supply [130,131].

This dysfunctional cellular network is sustained by a pathologically altered acellular milieu [132,133]. A defining feature is the toxic biochemical environment saturated with the SASP and pathological EVs, which collectively perpetuate inflammation and disrupt intercellular communication [124,134,135]. Concurrently, the physical structure of the ECM degrades due to SASP-secreted proteases. This leads to detrimental changes, such as altered collagen cross-linking and the accumulation of advanced glycation end products (AGEs), which increase matrix stiffness and, in turn, can further induce cellular senescence, thereby creating a potent vicious cycle [136,137]. This environment is also marked by profound metabolic distress, characterized by elevated ROS that contribute to widespread oxidative damage [138].

Functionally, these components synergize to create a state of chronic, low-grade inflammation (inflammaging) that impairs the regenerative capacity of the stem cell niche [139,140]. In essence, the SBM is a self-reinforcing pathological network that systematically dismantles skeletal homeostasis. Understanding its multifaceted nature is paramount, as it not only elucidates the complexity of age-related bone pathology but also provides the theoretical rationale for designing advanced biomaterials that can execute multi-pronged interventions to disrupt this environment and restore regenerative function [[141], [142], [143]].

## 3 Bone biomaterials for senescent cell elimination

The accumulation of senescent cells is a recognized driver of age-related bone loss and impaired regeneration, establishing their targeted elimination as a promising therapeutic strategy [[144], [145], [146]]. Although senolytic agents that selectively induce apoptosis by targeting anti-apoptotic pathways have shown remarkable potential in preclinical studies [32,[147], [148], [149], [150], [151]], their clinical translation faces core obstacles rooted in poor pharmacokinetics and a lack of target specificity [152,153].

For leading candidates like Dasatinib and Quercetin (D + Q), their low aqueous solubility and poor bioavailability, compounded by the physiological barrier of bone tissue, hinder the achievement of therapeutic concentrations at the target site [[154], [155], [156]]. Increasing systemic dosage to compensate for this inefficient delivery inevitably triggers severe class-wide off-target effects, including dose-limiting toxicities like thrombocytopenia [157,158]. Furthermore, for combination therapies like D + Q, maintaining a precise synergistic drug ratio at the target site is nearly impossible with conventional administration. These issues underscore the two fundamental hurdles of localizing senolytic agents to the target site and controlling their subsequent release. To address this fundamental delivery problem, biomaterial-based systems have been developed, aiming to re-engineer the therapeutic window of senolytics and transform them into spatially and temporally precise agents (Table 2) [159].

**Table 2:** Design strategies and biological effects of bone biomaterials for senescent cell elimination.

Material System	Key Design Strategy	Biological Function/Goal	Key Outcomes	Ref.
Quercetin-loaded phenylboronic acid-modified hydrogel	pH-responsive drug release	Responsive drug release in the acidic microenvironment of IVD degeneration to clear senescent NP cells	Effectively mitigated NP cell senescence, maintained disc structure, and alleviated pain sensitivity in a rat IVDD model	[160]
Quercetin-loaded MMPs-responsive hydrogel)	Senescent microenvironment-responsive release	Utilizing MMPs from senescent cells for local, responsive drug release at the bone defect	Effectively cleared senescent cells in the defect area, accelerating bone defect repair in aged rats	[161]
Peptide-modified Liposome	Active Bone Targeting	Targeted delivery of senolytics to bone tissue	Effectively cleared senescent cells in bone, promoted bone formation	[162]
Alendronate-modified drug-loaded liposome	Active Bone Targeting	Utilizing alendronate to target bone for co-delivering two senolytics at a precise ratio	Effectively cleared senescent cells in bone of chemo/radiotherapy-induced osteoporosis models, and improved bone mass and microstructure	[163]
Aptamer-functionalized drug-loaded liposome	Aptamer-mediated cell-specific targeting	Using an aptamer to specifically target synoviocytes to clear senescent FLSs in OA joints	In a mouse OA model, effectively cleared senescent cells in the synovium and attenuated cartilage degradation	[164]
Prodrug-loaded, peptide-modified EVs	Active bone targeting combined with intracellular enzyme response in senescent cells	Targeting a prodrug to bone for specific activation within senescent osteocytes to selectively clear them	Selectively cleared senescent osteocytes in aged mice, mitigating age-related bone loss with no obvious toxicity	[173]
Immunoliposome and peptide-loaded hydrogel microspheres	Stage-specific precision regulation of senescent cells: reversing early-stage, clearing late-stage	Specifically targeting and reversing early senescent cells while selectively clearing late senescent cells	Effectively reversed early senescent cell function and cleared late senescent cells, significantly alleviating rat IVD degeneration	[176]
Drug-loaded aldehyde-modified HA microspheres	Inducing senescence apoptosis and in situ remodeling of efferocytosis	Targeting degraded cartilage, clearing senescent cells to reverse cartilage senescence	Effectively cleared senescent chondrocytes, reversed senescence, and promoted cartilage repair in an OA model	[177]
Quercetin-loaded aligned nanowire hydrogel	Synergy of biological cues (senolytic) and topographical cues (aligned structure)	Synergistically mitigating stem cell senescence while guiding osteo/tenogenic differentiation to promote tendon-to-bone healing	Effectively mitigated stem cell senescence, promoting repair and functional recovery of the tendon-to-bone interface in an osteoporotic rat model	[159]
Photo-activatable immunomodulatory exosome	Active targeting of senescent cells and photo-activation of NK cells	Activate NK cells to selectively clear senescent FLS	Activated the cGAS-STING pathway in NK cells, enhancing senescent cell clearance, effectively delaying OA progression	[183]

### 3.1 Microenvironment-responsive elimination strategies

**Fig. 3:**
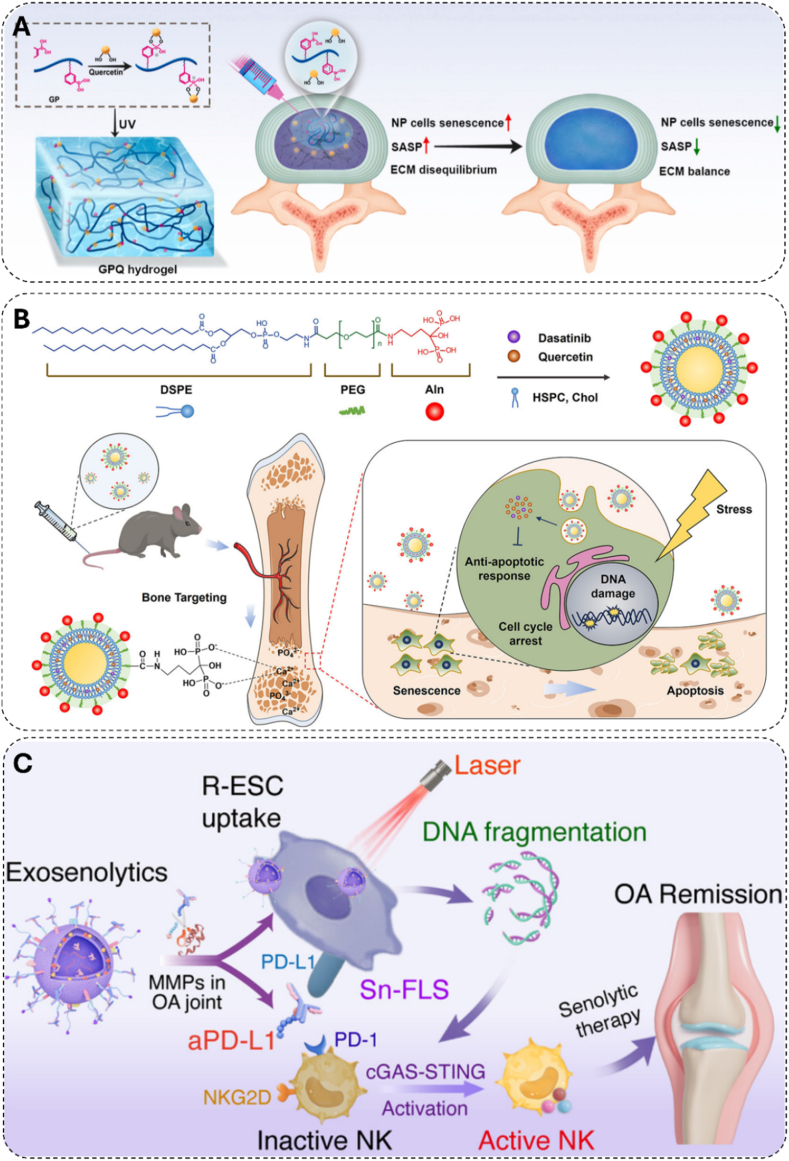
Biomaterial-based strategies for senescent cell elimination in bone tissue. A) Schematic of GPQ hydrogel and its application in degenerative IVD treatment. Copyright 2024, KeAi Communications Co., Ltd [160]. B) Schematic depiction of liposomes functionalized with alendronate for osseous tissue-targeted delivery of senolytic drugs to eliminate senescent cells. Copyright 2024, The Author(s) [163]. C) Light-inducible senolytic drugs promote NK cell activation for OA prevention via cGAS/STING pathway stimulation. Copyright 2025, American Chemical Society [183].

Addressing these delivery challenges, a new generation of intelligent biomaterials has emerged, evolving from strategies that control when the drug is released to those that dictate where and how it is activated. The initial approach centered on environment-responsive systems, which represent a paradigm shift from passive diffusion to on-demand release. The senescent microenvironment exhibits biochemical characteristics including inflammation-induced acidic pH and elevated matrix metalloproteinases (MMPs) secreted as part of the SASP. Initial strategies exploited pH changes as triggers. Wang et al. developed a hydrogel system utilizing boronate chemistry by grafting phenylboronic acid groups onto gelatin methacrylate and incorporating quercetin through reversible boronate bonds (Fig. 3A). Under physiological conditions (pH 7.4), the boronate ester bonds remain stable; in acidic microenvironments (pH < 6.5), these bonds decompose, triggering quercetin release. In vitro studies showed downregulation of senescence markers p16 and p21 in nucleus pulposus (NP) cells, while single local injection in rat intervertebral disc degeneration (IVDD) models yielded improved therapeutic outcomes after 8 weeks, evidenced by enhanced MRI signal intensity and improved Pfirrmann grading [160].

While pH-responsive systems provided environmental sensitivity, their targeting precision remained limited by non-specific pH gradients. This limitation drove development of materials responsive to senescent cell-specific molecular signatures. Xing et al. engineered a SASP-responsive TG-18 hydrogel that self-assembles into ordered fibrous networks and degrades specifically in response to senescent cell-secreted MMPs. This enzymatic degradation enables targeted quercetin release, efficiently clearing senescent cells while minimizing off-target effects. The MMP-responsive approach represents advancement from environmental sensing to cellular signal recognition, demonstrating effective elimination efficiency and bone regeneration in aged rat models [161].

### 3.2 Multi-scale spatial control: from tissue-level to cell-specific delivery

To shift the precision of senolytic therapy from environmental responsiveness to spatial specificity, researchers have engineered targeted delivery systems that can dictate the location of drug release. Bone-specific delivery emerged as a critical strategy given the unique challenges of drug penetration into mineralized tissue. Xing et al. developed (AspSerSer)_6_ peptide-modified liposomes that exploit the high affinity of aspartic acid-serine-serine sequences for hydroxyapatite in bone matrix [162]. This bone-targeting approach achieved enhanced quercetin accumulation in femoral and tibial tissues, effectively eliminating senescent BMSC while minimizing systemic exposure. Building upon this foundation, Li et al. created alendronate-functionalized liposomes co-loaded with dasatinib and quercetin (Aln-Lipo-DQ) to address the critical challenge of maintaining optimal drug ratios at target sites (Fig. 3B) [163]. The alendronate modification provided superior bone mineral affinity, increasing bone volume/total volume (BV/TV) from 5.05 % to 11.95 % in chemotherapy-induced osteoporosis models while achieving 2.91-fold improvement in radiation-induced bone loss compared to conventional delivery.

Beyond tissue-level targeting, the next challenge is achieving cellular specificity within designated tissues. Aptamer technology offers a solution for cell-specific drug delivery. Chen et al. developed aptamer CX3 through cell-SELEX technology, demonstrating high specificity for fibroblast-like synoviocytes (FLS) with nanomolar binding affinity (Kd = 24.6 ± 3.5 nM) while showing negligible interaction with chondrocytes. CX3-functionalized liposomes (CX3-LS-DQ) achieved 1.4-fold enhanced accumulation in synovial regions compared to conventional liposomes, with substantially reduced off-target distribution. This targeting precision was corroborated at the cellular level, where the system demonstrated excellent biocompatibility, maintaining over 90 % viability in non-target chondrocytes and healthy synoviocytes while selectively eliminating their senescent counterparts. In DMM-induced osteoarthritis (OA) models, CX3-LS-DQ effectively eliminated senescent synovial fibroblasts, suppressed SASP factor secretion, and significantly preserved cartilage integrity with Osteoarthritis Research Society International (OARSI) scores reduced by approximately 70 % [164].

### 3.3 Senescence-selective activation and modulation

While second-generation platforms achieve cell-type specificity through targeting particular cellular populations, they cannot distinguish senescent from healthy cells within the same cell type. Third-generation strategies address this limitation by exploiting senescence-specific signatures to selectively target cells based on their senescent state rather than their cellular identity. The inaugural approach emerged from exploiting elevated SA-β-gal activity, a hallmark feature of senescent cells [32,[165], [166], [167], [168], [169], [170], [171]]. Agostini et al. pioneered this concept by developing galactose oligosaccharide-modified mesoporous silica nanoparticles that established responsive drug release specifically activated within senescent cell lysosomes [172].

Building upon this foundation, He et al. advanced the approach by engineering a sophisticated dual-targeting platform that combined metabolic recognition with spatial precision [173]. Their system employed a galactose-modified maytansinoid prodrug (DM1-Gal) that remains inactive in normal cells but undergoes hydrolysis by SA-β-gal in senescent cells, releasing active DM1 selectively. This activation mechanism demonstrated superior senolytic specificity compared to free drug administration. To achieve bone tissue accumulation, they engineered macrophage-derived EVs with (AspSerSer)_6_ peptide modifications. This design created a triple-specificity delivery system that integrated tissue targeting, cellular recognition, and enzymatic activation. The (AspSerSer)_6_-sEV/DM1-Gal system entered cells through clathrin-mediated endocytosis and achieved selective activation only in senescent osteocytes. Upon activation, DM1 disrupted microtubule assembly, leading to AKT destabilization and apoptotic activation through Bax/Bcl-2 modulation and caspase-3 activation. In aged mouse models, this approach effectively eliminated senescent osteocytes while preserving normal bone cells, achieving improved bone parameters and reduced age-related bone loss with minimal systemic toxicity. This intelligent activation strategy represents advancement toward achieving senescent cell specificity while protecting normal cellular populations. However, β-galactosidase activity can increase in non-senescent cells under stress conditions, indicating the need for recognition mechanisms with greater precision [174,175].

While such intelligent activation strategies represent a key milestone in the selective elimination of senescent cells, the frontier of precision is already expanding from targeting specific cells to tailoring therapeutic interventions. This evolution from uniform clearance to state-dependent clearance strategies is driven by the growing understanding of senescence heterogeneity. This conceptual shift is well illustrated by Xiang et al., who engineered a HAMA/FOXO4-DRI/Ab-Lipos system designed to modulate cell fate through stage-specific interventions [176]. This system achieves a dual function wherein AQP1 antibody-functionalized liposomes rejuvenate early-stage senescent cells by restoring mitochondrial function, and an embedded FOXO4-DRI peptide selectively eliminates late-stage counterparts by reactivating p53-mediated apoptosis. The demonstrated success of this approach in reversing IVDD marks a pivotal advance in senolytic precision, providing a proof-of-concept for state-dependent therapies that can discriminate between cellular states to selectively eliminate pathogenic cells while actively preserving functional ones.

In summary, the evolution of biomaterials for senescent cell elimination from simple delivery vehicles to sophisticated, cell-state-specific platforms marks a remarkable progression in precision. However, strategies predicated on inducing cell death are confronted by the fundamental biological challenge of ensuring the efficient clearance of apoptotic cells. Within the aged or pathological bone microenvironment, impaired efferocytosis can lead to the accumulation of cellular debris and secondary inflammation, potentially undermining therapeutic benefits [[177], [178], [179]]. This very challenge of post-elimination clearance, compounded by the limitations of direct pharmacological killing, is driving a strategic pivot toward integrated, immunomodulatory paradigms [180,181].

### 3.4 Immuno-modulatory strategies for senescent cell clearance

To address the challenge of inefficient clearance, this paradigm shifts the function of biomaterials from direct cytotoxic agents to immunomodulatory platforms. These are designed to reactivate and amplify the host's own immune system for a more effective and physiologically relevant elimination of senescent cells [182]. The research by Zhang et al. epitomizes this nascent strategy [183]. They constructed photoactivatable exosenolytics using macrophage-derived exosomes co-modified with senescent cell-targeting ligands (RGD) and an immune checkpoint inhibitor (aPD-L1). Encapsulating a photosensitizer and an NKG2D ligand activator, the system not only induces a local immunogenic response via photodynamic therapy but also potentiates the recognition, adhesion, and cytotoxicity of Natural Killer (NK) cells against FLS by activating the cGAS-STING pathway (Fig. 3C). This immuno-sensitization approach successfully integrates exogenous intervention with endogenous immune remodeling, representing a significant advance in immune-mediated killing. Despite this leap in spatial control, clinical translation will demand even more sophisticated designs. Ensuring long-term safety by mitigating the inherent risks of autoimmune reactions [184] and cytokine release syndrome [185] remains a central challenge for the next generation of immunomodulatory biomaterials.

Collectively, these strategies demonstrate a clear trajectory toward precision senolysis, advancing from nonspecific environmental triggers to sophisticated multi-scale platforms capable of tissue-, cell-, and cell-state-specific targeting, culminating in immunomodulatory approaches that harness endogenous clearance mechanisms. This increasing sophistication in controlling both drug delivery and therapeutic action helps mitigate the systemic toxicity and poor bioavailability that have hindered clinical translation of conventional senolytic agents. Such precision-driven strategies are therefore paramount for developing clinically viable therapies with enhanced safety and efficacy for age-related bone diseases.

## 4 Bone biomaterials for senescent cell rejuvenation

Recent studies have illuminated the dual role of senescent cells in tissue homeostasis. While contributing to aging-related pathologies, they also facilitate wound healing and tissue repair through temporally regulated secretory activities [186,187]. This mechanistic understanding has catalyzed the development of sophisticated biomaterial-based strategies that aim to modulate senescent cell activities rather than eliminate them, thereby preserving their regenerative potential while attenuating detrimental effects [24,[188], [189], [190]]. Achieving this targeted rejuvenation requires interventions that address the core pillars of the senescent phenotype. Such strategies operate on multiple biological scales, progressing from downstream consequences to upstream causes. One approach involves restoring inflammatory homeostasis to resolve the chronic sterile inflammation of the SASP. A deeper intervention aims to reverse metabolic dysfunction, thereby interrupting the deleterious feedback loop between mitochondrial decay and oxidative stress. The most foundational strategies seek to reprogram the dysregulated gene expression that orchestrates the senescent state. This chapter details biomaterials designed to execute these distinct yet interconnected strategies, offering precise therapeutic interventions for age-related bone disorders by fundamentally remodeling senescent cell function [191] (Table 3).

**Table 3:** Design strategies and biological effects of bone biomaterials for senescent cell rejuvenation.

Strategy/Material System	Key Design Strategy	Biological Function/Goal	Key Outcomes	Ref.
**Restoring Inflammatory Homeostasis**				
Ruxolitinib-loaded PCL/BG nanofibers	Local sustained release of JAK inhibitor	Inhibit SASP and repair the SBM	Effectively suppressed senescent phenotype, promoted aged bone regeneration	[196]
Metformin-loaded strontium alginate hydrogel	Synergistic anti-inflammatory strategy integrating a bioactive ion (Strontium) with a loaded drug (Metformin)	Suppress the pro-inflammatory phenotype of senescent cells	Significantly downregulated the expression of inflammation and senescence-related genes	[197]
Chitosan-strontium chondroitin sulfate (CH-SrCS) porous scaffold	Leveraging the intrinsic bioactivity of the biomaterial components	Inhibit inflammatory responses and osteoclastogenesis	Downregulated pro-inflammatory and osteoclastogenic genes, and promoted bone regeneration in aged rats	[198]
Metformin-loaded sulfobetaine (SB)-modified hyaluronic acid microspheres	Dual-mechanism: Integrating physical lubrication for stress reduction with drug release for anti-inflammation	Simultaneously targeting inflammation induced by both mechanical stress and biochemical signaling	Significantly enhanced joint lubrication and effectively reversed senescent phenotypes and OA progression in aged mice	[199]
**Restoring Metabolic Function**				
RSV-loaded ZIF-8	pH-responsive delivery	Responsive delivery of antioxidant (RSV) to scavenge ROS	Effectively scavenged ROS, restored osteogenic and angiogenic functions of senescent cells	[208]
Ultrasmall ZIF-8-coated SOD	Size-mediated intracellular delivery	Deliver antioxidant enzyme (SOD) bypassing lysosomes to scavenge intracellular ROS	Avoided lysosomal degradation, efficiently cleared intracellular ROS, significantly improved bone density	[209]
CeO_2_-infused PCL nanofibers	Intrinsic nanozyme catalysis	Local and sustained ROS scavenging to restore senescent cell function	Effectively scavenged ROS, restored osteogenic differentiation and mineralization in senescent cells	[210]
PAA-modified CeO_2_ nanoparticles (PCNPs)	Nanozyme activity & signaling pathway modulation	To scavenge ROS and restore mitochondrial function to alleviate senescence	Restored mitochondrial function, alleviated senescence, promoted diabetic rat bone regeneration	[211]
N-acetylcysteine-derived, iron-doped Carbon Dots (NAC-CDs)	Multi-enzyme mimicry & surface functionalization	To efficiently and broadly scavenge multiple ROS types to inhibit oxidative stress	Efficiently cleared ROS, inhibited senescence, and reversed in vivo disc degeneration	[212]
Composite system of diselenide nanocarriers and a carboxyl-rich hydrogel	Synergistic multi-compartment (intra-/extra-cellular) regulation	To synergistically scavenge intra- & extra-cellular ROS and enhance autophagy for rejuvenation	Significantly reduced senescent cells and increased new bone formation	[213]
Kremen1-targeting co-delivery nanomicelles of RSV/NR	Active targeting of senescent cells & synergistic drug co-delivery	“Activate and refuel” to synergistically enhance the SIRT1 pathway	Increased NAD + levels, activated SIRT1 pathway, promoting aged bone regeneration	[216]
Tea polyphenol-reduced graphene (TPG) composite hydrogel	Passive targeting with mitochondria-affinity material	To target mitochondria and activate the SIRT1/PI3K/AKT pathway	Reduced senescent cells and promoted bone regeneration via SIRT1 activation	[217]
Berberine-loaded chitosan nanoparticles (BBR-CNPs)	Sustained release and delivery of active molecules	To activate the SIRT3/SOD2 signaling axis to maintain mitochondrial function	Inhibited chondrocyte senescence and delayed OA progression	[218]
Ultrasound-responsive signal-transducing hydrogel microspheres	Physical signal transduction (ultrasound-to-heat)	To stabilize outer mitochondrial membrane via mild hyperthermia	Inhibited ROS and mtDNA leakage, mitigating inflammation and increasing bone density	[219]
Melatonin-functionalized mesoporous bioactive glass	Induction of tunneling nanotube (TNT) formation	To facilitate intercellular transfer of healthy mitochondria	Restored mitochondrial function and accelerated aged rat bone regeneration	[220]
α-KG-modified cerium-based nanosystem (CNS)	Dual-action system activates autophagy in senescent macrophages	Promote mitochondrial biogenesis and subsequent intercellular transfer to stem cells	Rejuvenated senescent stem cells and enhanced bone regeneration in vivo	[221]
ROS-responsive hydrogel delivering engineered microvesicles (iMVs)	Smart and sustained release of paracrine signals	To regulate mitochondrial dynamics to delay cellular senescence	Regulated mitochondrial dynamics, effectively ameliorating chondrocyte senescence	[222]
Energy metabolism-engaged nanomedicine (EM-eNM)	Biomimetic design as structural analog of inorganic polyphosphate (polyP)	Bind ATP5B, induce fission and mitophagy, and restore stemness	Completely reversed osteoporosis and restored endogenous BMSCs stemness in vivo	[223]
**Restoring Gene Regulation**				
DNA tetrahedron-miR-21-5p nanocomplex	Nucleic acid protection and delivery via DNA self-assembly	Replenish downregulated miR-21-5p in senescent cells, restoring osteogenic function	Promoted osteogenesis in senescent cells and repaired aged rat bone defects	[227]
NPPC-targeting LNPs @ GAG hydrogel	Active targeting delivery with inflammatory microenvironment regulation	Deliver Klotho circRNA to rejuvenate senescent progenitor cells	Rejuvenated progenitor cells, reversing aging-associated IVDD	[228]
Surface-modified Hollow Mesoporous Silica Nanoparticle, HMSN	Senescence-responsive payload release	Targeted siRNA release in senescent cells to silence pathogenic ADAM19	Efficient gene knockdown in senescent cells, promoting cartilage regeneration	[229]
Zoledronate-modified bone-targeting LNPs	Bypassing endogenous post-transcriptional modification defect	Deliver m7G-modified mRNA, bypassing cellular translation defects	Restored bone formation in defect models, validating the strategy's efficacy	[230]
Magnesium-containing bioceramic (Akermanite, Akt)	Magnesium ions from material degradation act as bioactive signals	Stimulate senescent cells to secrete therapeutic miRNA-enriched exosomes	Induced secretion of miR-196a-5p-rich exosomes, promoting osteogenesis	[233]
Osteoinductive EV (oi-EV) @Mesoporous Bioactive Glass (MBG) scaffold	Material's hierarchical pore structure for EV protection and sustained release	Provide a long-term local supply of therapeutic EVs for aged bone repair	Achieved sustained EV release over two weeks, promoting aged rat bone regeneration	[234]
Dual-function engineered EV@GelMA hydrogel	Sequential functionalization: surface for recruitment, cargo for instruction	Sequentially recruit senescent stem cells and then instruct their osteogenesis	Successfully orchestrated the sequential function, outperforming single-function systems	[235]
Chondrocyte-targeting EVs@Thiolated hyaluronic acid (HA-SH) hydrogel	Biphasic release system via physical entrapment and chemical tethering	Achieve programmed, biphasic EV release to rejuvenate senescent chondrocytes	Achieved initial burst and long-term sustained release, effectively repairing OA cartilage	[236]

### 4.1 Biomaterial-mediated restoration of inflammatory homeostasis

During skeletal aging, the SASP serves as a critical mediator that impairs bone regeneration and remodeling through complex molecular networks, particularly the NF-κB, JAK-STAT, and mTOR signaling pathways [147,189,192,193]. Targeting the SASP-mediated inflammatory cascades is a primary strategy for cellular rejuvenation. Interventions designed to suppress the detrimental secretome without inducing cell death are often described as senomorphic, and engineering anti-inflammatory biomaterials to achieve this effect has emerged as a promising therapeutic avenue for age-related bone disorders [20,22,194,195]. Insights into these molecular mechanisms have guided the design of biomaterials, ranging from targeted intervention of specific signaling pathways to the development of multifunctional integrated anti-inflammatory systems.

**Fig. 4:**
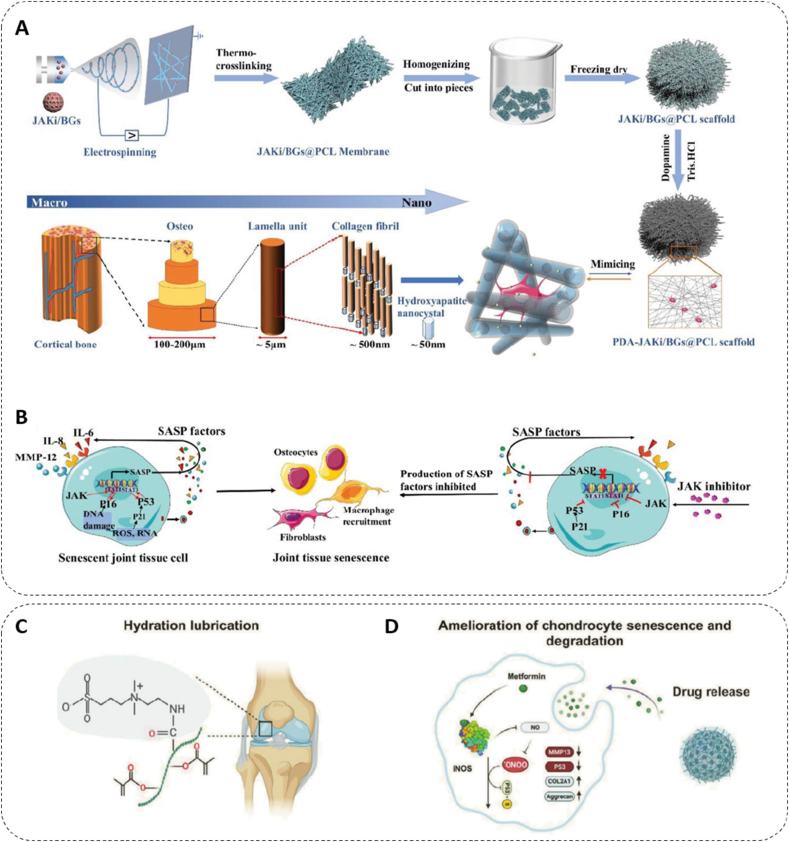
Anti-inflammatory bone biomaterials and mechanisms for senescent cell rejuvenation. A) Design and characterization of biomimetic ECM 3D nanofibrous scaffold. B) Mechanism of cellular senescence in joint tissue: stress-induced SNC activation through DNA damage, ROS, and aging. Copyright 2023, Wiley [196]. C, D) The molecular mechanisms by which Met@SBHA attenuates chondrocyte senescence and augments hydrated lubrication, consequently ameliorating age-associated OA pathogenesis. Copyright 2024, Wiley-VCH [199].

The initial exploration of anti-inflammatory biomaterials focused on modulating individual signaling pathways. The JAK/STAT signaling axis, crucial in cytokine secretion and inflammatory regulation, has been identified as a key target for reducing inflammation in aging bone tissue [193]. Liu et al. demonstrated this concept through an engineered scaffold system, where JAK1/2 inhibitor Ruxolitinib was encapsulated within mesoporous bioactive glass nanoparticles (BGs) and distributed throughout electrospun polycaprolactone (PCL) nanofibers (Fig. 4A). The addition of polydopamine (PDA) surface modification created a biomimetic scaffold with hierarchical architecture. This designed system demonstrated remarkable therapeutic outcomes through precise SASP regulation. In vitro studies revealed substantial suppression of SASP-related genes (IL-6, IL-8, MMP13) and inflammatory factors (Fig. 4B), while also reducing senescence signatures with SA-β-gal positive cells decreasing from 41.8 % to 23.1 %. When evaluated in a senescent rat calvarial defect model at 12 weeks post-implantation, micro-CT analysis revealed a significantly reduced structural model index, indicating the successful restoration of bone architecture to a more youthful state [196]. Similarly, Xu et al. employed metformin as an NF-κB pathway inhibitor within a sodium alginate/strontium composite hydrogel, effectively suppressing pro-inflammatory cytokine production from senescent cells while providing structural support for regenerating tissue [197].

An evolution of this pathway-targeted approach utilizes biomaterials with intrinsic bioactivity, eliminating the reliance on loaded drugs. For instance, Xu et al. developed a porous scaffold from chitosan and strontium chondroitin sulfate (SrCS) [198]. In this system, the chitosan and chondroitin sulfate components exert anti-inflammatory effects, while strontium (Sr) ions suppress osteoclast differentiation by modulating the NF-κB pathway. In vitro, the scaffold suppressed the expression of pro-inflammatory and osteoclastogenic genes (IL-1β, MMP9, CTSK) in macrophages and IL-6 in pre-osteoblasts. Concurrently, it upregulated the pivotal osteogenic gene BMP2. When evaluated in a femoral defect model using aged rats, the scaffold significantly enhanced bone regeneration. After four weeks, the treated defects exhibited more than double the BV/TV and significantly higher BMD compared to untreated controls. This work showcases a material-intrinsic strategy of suppressing inflammation to guide tissue regeneration in the aged skeleton.

While targeting these biochemical pathways is effective, a more comprehensive anti-inflammatory strategy must also account for the physical triggers of inflammation. Hou et al. advanced this concept by addressing the multifaceted nature of senescence-associated inflammation. They recognized that in mechanically active tissues, physical stressors are themselves potent inflammatory stimuli. To address both the mechanical and biochemical origins of inflammation, they engineered a sophisticated microsphere system based on sulfobetaine-modified hyaluronic acid methacrylate. This biomimetic design integrates zwitterionic surface chemistry for exceptional boundary lubrication with metformin loading. This dual-function design allowed the system to simultaneously prevent mechanically-induced inflammation at the tissue interface while modulating the intrinsic iNOS/ONOO-/P53 signaling axis in senescent cells via metformin release (Fig. 4C and D). When applied intra-articularly in aged mice, this integrated anti-inflammatory system effectively reversed inflammatory phenotypes and facilitated tissue regeneration, demonstrating how a mechanistically synergistic approach can redirect senescent cells from pathological to regenerative functions [199].

The gradual evolution from single-pathway inhibitors toward more integrated anti-inflammatory biomaterial systems suggests a promising direction in addressing senescence-associated bone disorders. Beyond merely targeting inflammatory pathways, future innovations may focus on temporally programmable biomaterials capable of responding to different phases of the senescence program, thereby preserving beneficial repair functions while inhibiting chronic inflammatory components. This shift from static anti-inflammatory interventions to dynamic, stage-specific modulation could offer more sophisticated therapeutic strategies for age-related bone degeneration.

### 4.2 Biomaterial-mediated restoration of metabolic function

The metabolic dysfunction of senescent cells represents a distinct therapeutic target [200]. Central to this dysfunction is a vicious cycle driven by mitochondrial decay and oxidative stress, which critically impedes aged bone repair [201,202]. Consequently, developing biomaterials that can interrupt this cycle is a central strategy for cellular rejuvenation and enhanced bone regeneration. This chapter focuses on two classes of metabolism-targeting biomaterials designed to restore redox homeostasis and repair mitochondrial function.

#### 4.2.1 Restoration of redox homeostasis

Restoring redox homeostasis is one of the most direct strategies to counteract metabolic dysregulation in senescent cells [203]. Its primary objective is to lower excessive intracellular ROS levels, thereby mitigating oxidative stress [204,205]. Biomaterial-based approaches are broadly categorized as either functional carriers for exogenous antioxidants or intrinsically active agents with endogenous, regenerative antioxidant capabilities.

As functional carriers, biomaterials can overcome the common drawbacks of conventional antioxidants, such as poor stability, short half-life, and low bioavailability [206,207]. Early strategies focused on targeted delivery through environment-responsive systems, such as the work by Zheng et al. who utilized pH-responsive ZIF-8 metal-organic frameworks to deliver resveratrol (RSV) [208]. Subsequent work employed more refined encapsulation techniques to improve delivery efficiency and protect biological activity. For instance, Liang et al. encapsulated the SOD enzyme within an ultrathin ZIF-8 coating, creating ultrasmall nanoformulations of only ≈9 nm. This size enabled direct penetration of the cell membrane, thereby bypassing the lysosomal degradation pathway and resulting in a BMD increment approximately twice that of native SOD in an aged osteoporosis model [209].

**Fig. 5:**
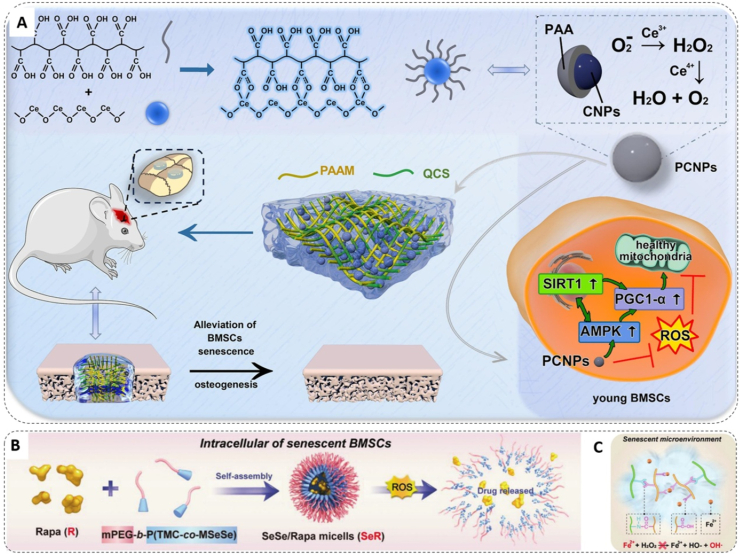
Antioxidative bone biomaterials and their mechanisms for senescent cell rejuvenation and enhanced bone regeneration. A) Schematic representation of PCNPs for rejuvenating BMSCs and promoting bone regeneration in type 2 diabetes mellitus (T2DM). Copyright 2024, Elsevier [211]. B) Synthetic pathway of mPEG-b-P(TMC-co-MSeSe) and SeR nanomicelles. C) Mechanism of PEGS-PGA in aging microenvironment regulation via carboxyl groups. Copyright 2023, Wiley-VCH [213].

An alternative strategy involves designing integrated biomaterials with intrinsic antioxidant activity. These materials act as ROS scavengers themselves, requiring no drug loading and offering more direct and sustained effects. Nanozymes, materials with enzyme-like activities, exemplify this strategy. Nilawar et al. constructed a dynamic antioxidative scaffold by combining cerium oxide (CeO_2_) nanoparticles, which leverage Ce^3+^/Ce^4+^ cycling, with PCL nanofibers to achieve sustained redox modulation in senescent osteoblasts [210]. Building on this, Wang et al. further optimized CeO_2_ nanoparticles, enhancing their multi-enzyme mimetic activities and were shown to restore mitochondrial function through the activation of the AMPK-SIRT1-PGC1α pathway (Fig. 5A) [211]. To address the limited catalytic diversity of single-metal nanozymes, Wu et al. developed a multi-enzyme mimetic system using N-acetylcysteine (NAC) as a precursor for iron-doped carbon dots. This system exhibited high SOD-like activity (≈250 U mg^−1^), and its optimized size and functionalized surface achieved over 90 % ROS elimination while effectively downregulating senescence markers like p21 [212].

While the aforementioned strategies primarily target intracellular ROS, recent research has expanded the regulatory scope to the extracellular space for more comprehensive oxidative stress management. He et al. designed a multi-compartmental regulatory system that integrates diselenide nanocarriers, which scavenge intracellular ROS while releasing rapamycin to enhance autophagy, with a carboxyl-rich hydrogel matrix that inhibits extracellular hydroxyl radical formation by sequestering Fe^2+^ (Fig. 5B and C). In aged mouse models, this dual-space approach reduced the proportion of senescent BMSCs at the defect site by nearly threefold and led to a ≈2.75-fold increase in new bone volume, highlighting the potential of this design in antioxidative biomaterial development [213].

In summary, the design of antioxidative bone biomaterials has progressed from passive delivery to active catalysis and multi-spatial regulation. Nevertheless, these strategies primarily address the consequences of oxidative stress, namely, scavenging ROS that have already been produced. A more fundamental approach is to target the cause. Given that dysfunctional mitochondria are the primary source of ROS in senescent cells, developing biomaterials that directly target and repair mitochondrial function offers a proactive strategy to resolve the issue at its origin, representing a key frontier in the field.

#### 4.2.2 Restoration of mitochondrial function

Directly targeting and repairing mitochondrial function offers a path to address the metabolic dysregulation of senescent cells at its source [75,214,215]. To this end, biomaterial strategies are being deployed across multiple scales of intervention, ranging from modulating cellular signaling pathways to remodeling mitochondrial networks and, more recently, directly targeting core molecular machinery within the organelle itself.

A primary strategy involves the modulation of critical signaling pathways, particularly the sirtuin (SIRT) family. The function of SIRT1 is often compromised in senescent cells due to low NAD + availability. To address this, Wang et al. developed a nanoplatform for the co-delivery of the SIRT1 activator RSV and the NAD + precursor nicotinamide riboside (NR). This system, which targeted the senescent BMSC marker Kremen1, increased intracellular NAD + levels approximately 17-fold and effectively delayed senescence through synergistic SIRT1 activation [216]. In another approach, Fu et al. designed a hydrogel that releases tea polyphenol-reduced graphene (TPG). The TPG material exhibits mitochondrial affinity and activates the SIRT1/PI3K/AKT pathway, reducing the proportion of senescent BMSCs in a bone defect model from approximately 46 %–3.5 % [217]. The focus also extends to mitochondria-resident sirtuins. For example, Zhang et al. used chitosan nanoparticles to deliver berberine (BBR), which activated the SIRT3/SOD2 signaling axis. This enhanced the activity of the crucial antioxidant enzyme SOD2, thereby preserving mitochondrial function and improving the functionality of senescent chondrocytes [218].

Beyond pathway modulation, emerging strategies remodel mitochondrial homeostasis using non-pharmacological methods. Physical stimulation presents one such avenue. Huang et al. constructed an ultrasound-responsive hydrogel that converts non-invasive ultrasonic signals into localized mild hyperthermia of approximately 42 °C. This thermal stimulus induces heat shock proteins that stabilize the outer mitochondrial membrane, which in turn inhibits both ROS and mtDNA leakage and mitigates downstream cGAS-STING pathway activation [219].

**Fig. 6:**
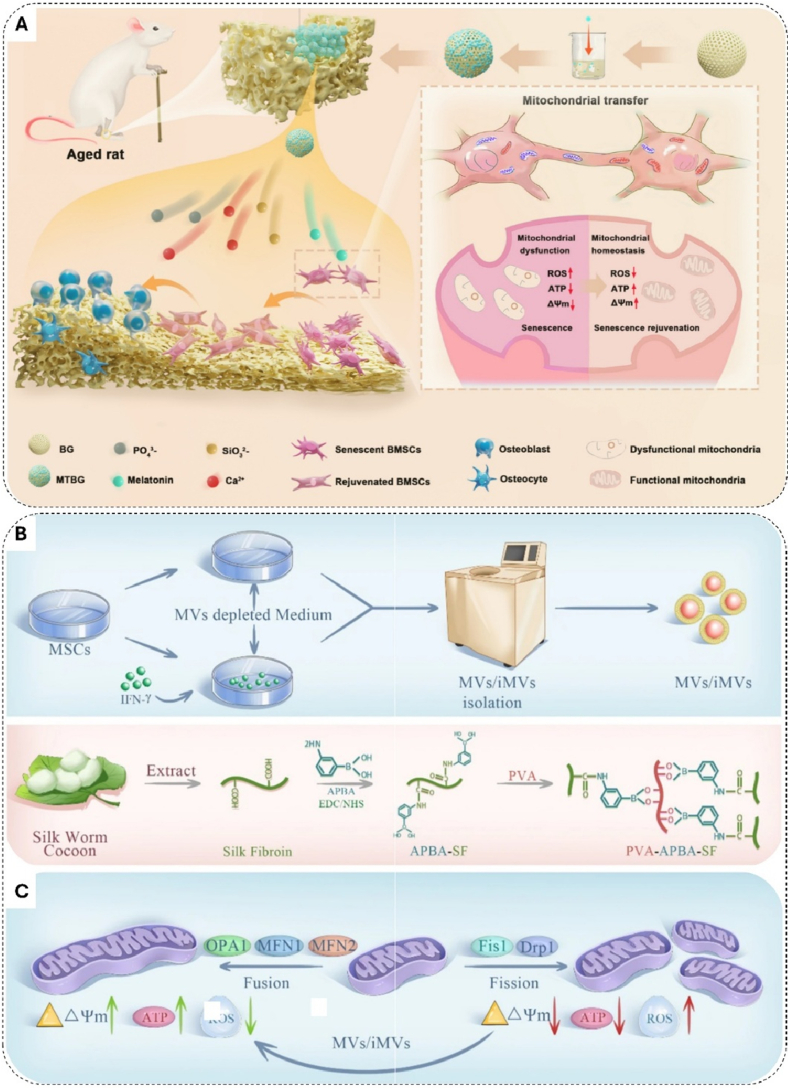
Mitochondria-targeting bone biomaterials for rejuvenating senescent cells and enhancing aged bone regeneration. A) Schematic of the proposed mechanism for accelerating aged bone regeneration via enhanced mitochondrial transfer mediated by tunneling nanotubes using MTBG. Copyright 2024, Elsevier [220]. B) Principle and production of Hydrogel@iMVs. MVs/iMVs are obtained through interventions and isolated by gradient centrifugation. Silk fibroin (SF), derived from silkworm cocoons, is synthesized into a responsive hydrogel as the carrier. C) Hydrogel@iMVs inhibit mitochondrial fission, promote fusion in chondrocytes, and prevent ROS-induced senescence. Copyright 2023, The Author(s) [222].

A conceptually distinct strategy operates at a structural level by facilitating the intercellular transfer of healthy mitochondria. Xiong et al. developed melatonin-functionalized bioactive glass microspheres that induce the formation of tunneling nanotubes (TNTs) (Fig. 6A), facilitating the direct transfer of mitochondria from healthy to senescent cells [220]. Building on this concept, Zhang et al. engineered a nanosystem that first rejuvenates senescent macrophages through autophagy-mediated mitochondrial biogenesis. These reprogrammed macrophages then serve as effective mitochondrial donors to senescent BMSCs [221].

Paracrine signaling also offers a means for functional reprogramming. Liu et al. used a ROS-responsive hydrogel for the sustained release of microvesicles from IFN-γ-prestimulated mesenchymal stem cells (iMVs) (Fig. 6B). These iMVs restored mitochondrial network homeostasis by upregulating fusion proteins OPA1 and MFN1 while inhibiting excessive fission, thus delaying cellular senescence (Fig. 6C) [222].

A more recent and highly precise strategy involves directly targeting molecular machinery within the mitochondria. Chen et al. developed an ‘energy metabolism-engaged nanomedicine’ (EM-eNM) based on ultra-small black phosphorus quantum dots. These were surface-engineered to function as a structural analog of inorganic polyphosphate (polyP). This biomimetic design facilitates their entry into mitochondria where they physically bind to the ATP5B subunit of the F1-ATP synthase complex. This material-based interaction inhibits excessive ATP synthesis, inducing a state that mimics energy restriction. Consequently, this triggered a cascade of endogenous quality control processes, including DRP1-mediated mitochondrial fission and subsequent PINK1/BNIP3-mediated mitophagy. This fundamental reprogramming shifted senescent BMSCs from oxidative phosphorylation toward a pro-stemness glycolytic state. The in vivo efficacy of this approach was substantial. Following systemic administration in 18-month-old mice, the EM-eNMs preferentially accumulated in bone and completely reversed age-related osteoporosis. Key metrics were restored to the levels of young controls, with BV/TV increasing from approximately 2.61 %–11.23 % and BMD from approximately 186 mg/cm^3^ to 567 mg/cm^3^ [223]. This work is notable for its use of a synthetic nanomaterial to directly interface with a core bioenergetic process.

In summary, biomaterial strategies for mitochondrial restoration show a clear progression from modulating cellular signaling to remodeling organellar networks and engaging intra-organellar molecular machinery. Future work will likely expand this biomimetic effector paradigm to other endogenous molecules and metabolic nodes. The potency of such direct interventions, however, raises critical questions regarding their specificity, potential off-target effects, and long-term fate. Therefore, enhancing the controllability of these therapeutic platforms will be essential for their translation.

### 4.3 Biomaterial-mediated restoration of gene regulation

The pathological phenotypes of cellular senescence, including inflammatory and metabolic dysregulation (4.1, 4.2), are driven by dysregulated gene expression programs. While direct intervention with therapeutic nucleic acids (e.g., miRNA, siRNA, mRNA) represents a fundamental strategy, their inherent instability and inefficient cellular uptake create significant translational barriers [224]. This section therefore reviews how bone biomaterials are engineered as delivery platforms to overcome these barriers, enabling the functional reprogramming of senescent cells by ensuring the protected, targeted, and controlled release of genetic cargo.

#### 4.3.1 Precision gene programming

A direct gene programming strategy for senescent cell rejuvenation involves replenishing critical regulatory molecules depleted during aging. This principle has been applied across different tissues using distinct nanodelivery platforms [225].

**Fig. 7:**
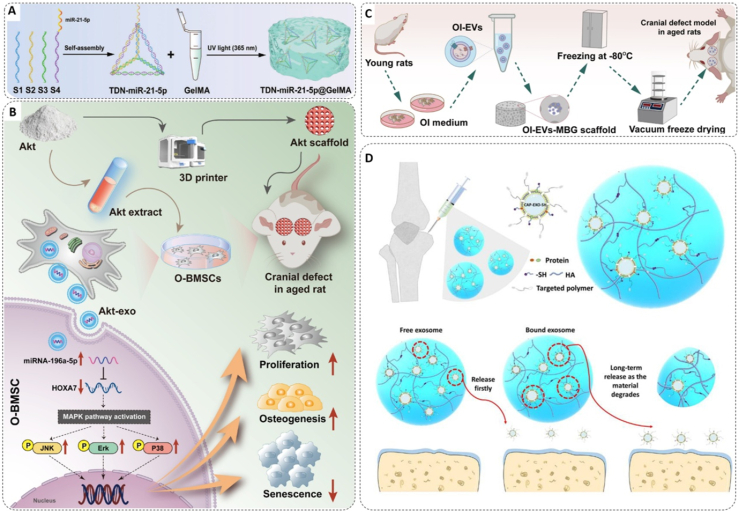
Bone biomaterials delivering therapeutic RNAs and EVs to target senescent cells and promote aged bone regeneration. A) Fabrication process of TDN-miR-21-5p nanocomplex. Copyright 2024, Wiley [227]. B) Mechanism of exosomal miR-196a-5p-mediated Akt activation in O-BMSC osteogenesis. Copyright 2024, KeAi Communications Co., Ltd [233]. C) Design and implantation of OI-EVs-MBG-gelatin scaffold in aged rat cranial defects. Copyright 2024, Elsevier [234]. D) Two-phase sustained release of exosomes from injectable HA-SH microgels: system design and release mechanism. Copyright 2023, American Chemical Society [236].

For instance, in bone regeneration, the reduced osteogenic capacity of senescent BMSCs is linked to downregulated miR-21-5p [226]. To counteract this, self-assembled DNA tetrahedra (TDNs) have been engineered as nanocarriers, whose defined nanostructure protects the miRNA cargo and facilitates efficient cellular uptake to restore osteogenic function (Fig. 7A) [227]. Following a similar logic in the context of related musculoskeletal disorders like IVDD, the decline of the anti-aging protein Klotho is a key driver of senescence in nucleus pulposus progenitor cells (NPPCs). To address this, researchers have utilized lipid nanoparticles (LNPs) to deliver Klotho-encoding circular RNA (circRNA), which successfully rejuvenated the progenitor cells and promoted ECM synthesis [228]. While this direct replenishment approach is effective, it represents a foundational strategy. A more advanced level of precision requires systems that do not just deliver a cargo, but first sense the pathological state of the target cell before releasing the therapeutic payload on-demand.

While restoring lost functions is one approach, a more proactive strategy is required when senescence itself is driven by aberrantly activated harmful genes. Applying this silencing strategy to OA, Wang et al. [229] addressed the significant overexpression of the ADAM19 gene in senescent chondrocytes. They demonstrated that silencing ADAM19 via siRNA not only attenuated senescence but also promoted a regenerative phenotype. The critical challenge of delivering this siRNA was solved by engineering a “sense-and-release” system using hollow mesoporous silica nanoparticles (HMSNs). Leveraging the established SA-β-gal responsive strategy, they functionalized the nanoparticle surface with a galactose-based polymer, making payload release dependent on the high intracellular SA-β-gal activity. This design achieved a gene knockdown of ≈75 % specifically within senescent cells. Highlighting the therapeutic potential of this approach, a single intra-articular injection of the nanosystem promoted significant hyaline cartilage regeneration in a post-traumatic OA mouse model.

More fundamental still are challenges where senescence compromises the cell's own molecular machinery, necessitating strategies that bypass the defect. Liu et al. confronted this issue by targeting a post-transcriptional processing failure in senescent BMSCs [230]. They found that decreased levels of the enzyme METTL1 impaired the essential N7-methylguanosine (m7G) modification of Runx2 mRNA, which is required for its stability and translation. This renders therapies using standard Runx2 mRNA ineffective. To circumvent this, the authors co-engineered the therapeutic and its carrier by synthesizing Runx2 mRNA pre-modified with the m7G cap and encapsulating it in bone-targeting LNPs. The strategy's necessity was confirmed in METTL1-deficient mice, where LNPs with normal mRNA failed to promote bone regeneration. In contrast, the engineered m7G-Runx2-mRNA-LNPs successfully restored bone formation.

#### 4.3.2 Regulation of intercellular genetic communication

Beyond programming individual cells, a higher-order strategy reprograms cell populations by regulating intercellular communication. This regulation targets the flow of genetic messengers, primarily EVs, to override pro-senescent signals and orchestrate a coordinated regenerative response [231,232]. Here, biomaterials advance from delivery vehicles to regulatory platforms, engineered to control the generation, cargo, and delivery of therapeutic EVs. By commanding these local gene information streams, such materials offer a promising strategy to reverse senescence for bone regeneration.

The most fundamental of these regulatory strategies involves using a biomaterial's inherent chemistry as an active trigger for endogenous communication. This principle is exemplified by the work of Qi et al., who utilized a magnesium-containing bioceramic, Akermanite (Akt) [233]. The degradation of the Akt ceramic provides a sustained release of magnesium ions (Mg^2+^), a stimulus that reprograms the paracrine function of senescent BMSCs. This reprogramming first induces an intracellular pro-regenerative state, marked by reduced senescence markers, and culminates in a therapeutic secretome of exosomes specifically enriched with miR-196a-5p. These therapeutic EVs subsequently act on recipient cells, where the miR-196a-5p cargo suppresses its target, Hoxa7, to activate the MAPK signaling pathway and drive osteogenesis (Fig. 7B). This entire pro-regenerative communication cascade, originating from the material's chemistry, was ultimately validated in vivo, where the 3D-printed Akt scaffold significantly enhanced bone regeneration in aged rats.

A more direct approach than stimulating endogenous signals is to supply EVs pre-loaded with complex genetic instructions designed to restore entire gene networks in senescent cells. However, the rapid in vivo clearance of EVs necessitates a delivery platform for sustained local release [124]. The work of Qi et al. addresses this by using a Mesoporous Bioactive Glass (MBG) scaffold as a protective depot for osteoinductive EVs (oi-EVs) (Fig. 7C) [234]. The scaffold's hierarchical pore structure shielded the oi-EVs, ensuring their release for over two weeks. This payload contained a key lncRNA that restored a pro-osteogenic ceRNA network in senescent cells. This synergistic approach proved highly effective, nearly doubling new bone volume in aged rat defects (∼16 % BV/TV) compared to the scaffold alone (∼9.5 % BV/TV). This study thus demonstrates how a material's architecture can create a local supply of potent genetic information, translating complex molecular network theory into a functional regenerative strategy.

A key challenge in communication regulation is commanding the temporal sequence of regeneration, which requires multi-scale systems that engineer both the EV messenger and its delivery platform. This can be achieved by programming the biological function of the EV itself. For instance, Qi et al. developed a dual-function EV with a surface aptamer (Apt19) for recruiting senescent BMSCs and an internal miRNA cargo (miR-376b-5p) to subsequently trigger their osteogenesis [235]. Delivered from a GelMA hydrogel, this system successfully orchestrated a “recruit-then-instruct” sequence in vivo, proving superior to systems with only a single function. Alternatively, the delivery platform can be programmed to command the release kinetics. Cao et al. demonstrated this with a “two-phase” hydrogel system designed to provide both an initial burst and a sustained, long-term release of chondrocyte-targeting EVs (Fig. 7D) [236]. This was achieved by physically entrapping some EVs for immediate action, while chemically tethering others via disulfide bonds for a slower, prolonged effect. Such programmable, multi-scale platforms, whether commanding biological function or delivery kinetics, allow for precise temporal control over the regenerative process. Achieving this level of control is a key objective in the current development of therapeutic biomaterials. However, the clinical translation of these approaches requires addressing the technical challenges associated with EVs, such as their inherent heterogeneity, variability in cargo loading, and functional instability in vivo [237].

Collectively, these strategies reveal the expanding role of biomaterials, evolving from passive carriers into sophisticated platforms that actively command the flow of intercellular genetic information for cellular rejuvenation.

In this chapter, the strategies for rejuvenating senescent cells have progressed from targeting downstream phenotypes like chronic inflammation and metabolic dysfunction to modulating gene expression programs through precision programming. Looking forward, the next frontier lies in moving beyond treating the consequences of senescence to addressing its primary origins, such as the accumulation of DNA damage. Initial studies have demonstrated the feasibility of this strategy, using self-powered, motion-responsive biomaterials for on-demand release of engineered EVs designed to correct specific DNA damage pathways [238]. Interfacing directly with the DNA damage response machinery using such biomaterials is therefore a promising direction, with the potential to yield therapies that prevent senescence rather than merely mitigating its effects.

## 5 Bone biomaterials for remodeling the senescent cell microenvironment

While many therapeutic strategies have focused on directly eliminating or rejuvenating senescent cells, another promising avenue explores the remodeling of their surrounding microenvironment. The finding that ECM from young donors can restore the regenerative potential of senescent mesenchymal stem cells [239,240] substantiates the view of the local niche not as a passive backdrop, but as an active participant in perpetuating the senescent phenotype. Furthermore, emerging evidence highlights that the senescent microenvironment is not monolithic, exhibiting significant spatial heterogeneity in its vulnerability to age-related inflammatory remodeling. A recent landmark study in Nature demonstrated this principle, revealing that adult skull bone marrow maintains resilient hematopoietic activity with limited SASP-associated inflammation, in stark contrast to the age-related decline observed in long bone marrow [241]. This discovery of a naturally occurring, SASP-resistant niche offers a powerful new paradigm. It suggests that the goal of microenvironment remodeling should not only be to counteract detrimental changes, but also to biomimetically replicate the protective features of these resilient niches.

Inspired by these natural protective mechanisms, the field has turned to engineering biomaterials that can reshape compromised environments and recreate similar pro-regenerative conditions in aging bone [242]. This chapter details these remodeling strategies, which progress from restoring the niche's fundamental chemical and electrical balance, to modulating its physical and mechanical cues, and finally to regulating the intercellular communication that governs the immune response (Table 4).

**Table 4:** Design strategies and biological effects of bone biomaterials for remodeling the senescent cell microenvironment.

Strategy/Material System	Key Design Strategy	Biological Function/Goal	Key Outcomes	Ref.
**Biochemical and bioelectric homeostasis restoration**				
Baghdadite Ceramic Scaffold	Ion release and pH regulation	Providing an anti-senescent chemical microenvironment	Reduced SASP expression, promoting bone regeneration in aged rats	[250]
Magnesium Boride/Alginate Hydrogel (MB-ALG)	Mild alkalization and ROS scavenging	Neutralizing the acidic and oxidative microenvironment	Restored senescent cell proliferation and disc function	[251]
Metformin-loaded Layer-by-Layer Nanotubes	Antioxidant drug release and ECM reconstruction	Reducing oxidative stress and remodeling ECM homeostasis	Reduced SASP and enhanced mitophagy, improving osseointegration	[252]
Mg^2+^-modified Black Phosphorus Conductive Hydrogel	Restoration of ion homeostasis (Mg^2+^, Ca^2+^)	Rebuilding the pro-angiogenic chemical signaling environment	Upregulated VEGFR2, promoting vascularized bone regeneration	[253]
Mg-Ce-MOF Scaffold	ROS scavenging and functional ion release	Ameliorating the oxidative and inflammatory microenvironment	Delayed BMSC senescence and promoted M2 macrophage polarization	[254]
SIRT3 Gene-engineered Hydrogel	Iron chelation and gene delivery	Simulating a hypoxic and immunomodulatory microenvironment	Promoted M2 macrophage polarization, enhanced aged bone regeneration	[256]
**Physical and mechanical modulation**				
Polyacrylamide Hydrogel (80 kPa)	Optimizing substrate stiffness to inhibit Piezo1	Mitigating the inflammatory microenvironment at the soft-hard tissue interface	Inhibited Piezo1-mediated inflammation, promoting aged osseointegration	[267]
Polyacrylamide Hydrogel (76 kPa)	Optimizing substrate stiffness (76 kPa)	Constructing a pro-regenerative osteo-immune microenvironment	Induced M2 polarization of senescent macrophages, promoting angiogenesis and osteogenesis	[268]
10 nm TiO_2_ Nanotube Arrays	Nanoscale topographical modulation	Providing a rejuvenating physical microenvironment	Activated YAP signaling, restoring osteogenic potential of S-MSCs	[270]
Anisotropic Hydrogel Microrods	Microscale geometric modulation	Reshaping the cytoskeleton-nucleus mechanotransduction environment	Delayed senescence by inhibiting cGAS-STING pathway via YAP/TAZ	[271]
Self-assembling Biomimetic Nanospheres	Interfacial biolubrication	Reducing friction and mechanical stress at the joint interface	Reduced friction coefficient, attenuating chondrocyte senescence and inflammation	[273]
High-mobility network hydrogel microspheres	Enhancing elastic deformation to dissipate stress	Modulating abnormal mechanical stress on chondrocytes	Mitigated mechanical stress-induced chondrocyte senescence	[274]
**Biological communication reprogramming**				
Hydrogen-releasing Scaffold	Local and sustained hydrogen (H_2_) release	Remodeling the pro-inflammatory senescent immune microenvironment	Induced macrophage M2 polarization and promoted endogenous cell recruitment	[278]
Smart H_2_S-releasing Hydrogel	NIR/Glucose dual-responsive H_2_S release	Disrupting the SASP-driven senescence cascade network	Inhibited SASP secretion and restored mitophagy	[279]
Nanozyme/Drug-releasing Hydrogel	Immunomodulation via STING pathway inhibition	Remodeling the senescence-associated inflammatory immune microenvironment	Inhibited cGAS-STING pathway and promoted M2 macrophage polarization	[280]
Hybrid Nanozyme 3D-printed Scaffold	Mitochondria-targeting nanozyme therapy	Remodeling the mitochondria-dysfunction-driven senescent microenvironment	Rejuvenated senescent cells and regulated macrophage polarization	[281]
Stem Cell-Homing Gene/Drug-delivery Hydrogel	Stem cell homing combined with anti-senescence gene/drug delivery	Establishing a stem cell homing niche and blocking senescence communication	Recruited MSCs and inhibited CCN1-mediated senescence signaling	[282]
E7-peptide/Exosome Functionalized “Climbing Scaffold”	Active recruitment and rejuvenation of autologous stem cells	Constructing a stem cell homing and regenerative microenvironment	Reduced SASP and promoted multi-stage osseointegration	[283]

### 5.1 Restoration of biochemical and bioelectric homeostasis

Age-related alterations in bone tissue frequently disrupt local microenvironmental chemistry and consequently limit regenerative capacity. Such disruptions may be linked to systemic low-grade metabolic acidosis [243,244]. Systemic acidosis, amplified at the bone matrix surface by processes such as osteoclast activation, exacerbates the local acidic milieu [245,246]. This local environment results in ionic dysregulation through the loss of critical ions like calcium and phosphate while concurrently promoting ROS generation and chronic oxidative stress [[247], [248], [249]]. These interconnected factors can synergistically inhibit cellular activity, disrupt signaling, and inflict molecular damage, thereby impairing tissue repair. Biomaterial-based interventions have been developed to counteract these chemical imbalances, evolving from passive environmental stabilization to integrated platforms that address multiple facets of the pathological niche.

An initial strategy involves using biomaterials whose inherent composition and dissolution properties passively restore a more favorable chemical environment. Baghdadite (Ca_3_ZrSi_2_O_9_) ceramics, for example, have been shown to maintain the surrounding fluid at a physiological pH of 7.3–7.5, which may help counteract age-related acidosis. The material's dissolution also releases Ca^2+^ and silicon ions, modifying the local ionic profile. In culture with senescent osteoblasts, Baghdadite substrates reduced the percentage of SA-β-gal-positive cells to 7 %, compared to 21 % observed on HA/TCP ceramics. This effect was accompanied by reduced SASP-driven pro-senescent paracrine signaling to neighboring cells [250].

**Fig. 8:**
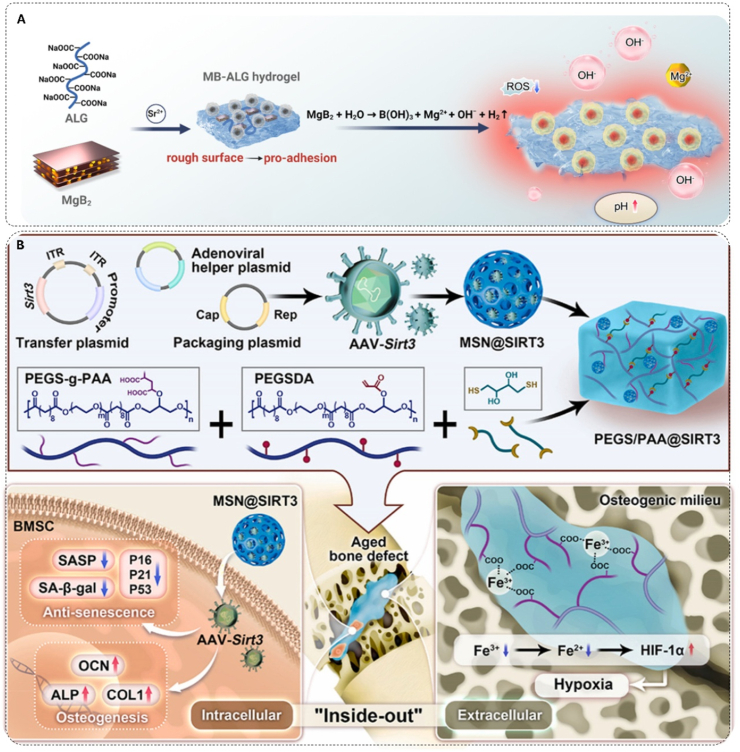
Biomaterial strategies for restoring biochemical and bioelectric homeostasis to promote functional bone regeneration. A) Schematic illustration of MB-ALG hydrogel preparation. Copyright 2024, American Chemical Society [251]. B) SIRT3-loaded hydrogel with “inside-out” strategy for aged bone regeneration. SIRT3 nano-vectors and PEGS/PAA polymer promote osteogenic differentiation and mimic hypoxic conditions to enhance bone healing. Copyright 2025, KeAi Communications Co., Ltd [256].

A more active approach involves designing materials to directly neutralize deleterious environmental components. To correct acidosis, hydrogels containing magnesium boride (MB) utilize a hydrolysis reaction that consumes protons, thereby elevating the local pH from an acidic state (pH ∼6.2) to a mildly alkaline one (pH ∼8.0) (Fig. 8A) [251]. To counteract oxidative stress, localized antioxidant delivery has been explored. Wu et al. used TiO_2_ nanotubes on a titanium implant for the sustained release of metformin [252]. This approach substantially lowered the oxidative stress burden in senescent mesenchymal stem cells and concurrently attenuated the expression of SASP factors, including IL-1β and TNF-α, in the surrounding milieu.

Integrated platforms aim to remodel multiple environmental facets simultaneously by combining several biochemical functionalities. Wu et al. developed a conductive hydrogel incorporating magnesium-modified black phosphorus (BP@Mg) to modulate the local electrochemical environment. The material's conductivity helps restore electrical homeostasis, while the sustained release of Mg^2+^ corrects ionic dysregulation [253]. Similarly, Sun et al. created a multifunctional metal-organic framework (Mg-Ce-MOF) that combines two modes of chemical remodeling [254]. The framework itself acts as a nanozyme, catalytically scavenging ROS through the Ce^3+^/Ce^4+^ redox cycle of its metal nodes and the antioxidant nature of its organic ligands. Concurrently, its degradation provides sustained release of Mg^2+^, reshaping the ionic milieu.

A Shifting the focus from neutralizing detrimental factors to proactively guiding cellular behavior, a more sophisticated strategy involves engineering the metabolic microenvironment. One prominent example is the regulation of oxygen tension, a critical determinant of cellular metabolic state and function [255]. Zhu et al. developed a gene-engineered hydrogel (PEGS/PAA@SIRT3) that actively creates a localized hypoxic niche by chelating free iron ions, which are essential for oxygen-consuming enzymatic reactions (Fig. 8B). This engineered hypoxia significantly upregulated HIF-1α expression in senescent cells, activating downstream regenerative signaling pathways [256]. Notably, this approach also restored cellular metabolic fitness by activating AMPK signaling, thereby enhancing glucose uptake and ATP synthesis. This metabolic rejuvenation recovered the compromised osteogenic potential of senescent cells and favored endochondral ossification.

The development of such multifunctional materials capable of orchestrating electrochemical, ionic, redox, and metabolic homeostasis represents the frontier in microenvironment engineering. The next critical challenge is to translate their complex, multifaceted functionalities into robust, predictable, and clinically viable therapies for treating age-related bone defects.

### 5.2 Physical and mechanical modulation

Beyond the chemical microenvironment, the physical and mechanical properties of the ECM serve as potent regulators of cell behavior through mechanotransduction [257]. Cells actively sense and respond to physical cues such as substrate stiffness and surface topography, which significantly influence their fate and function [[258], [259], [260]]. In aging tissues, the native ECM often undergoes stiffening and structural degradation, sending aberrant mechanical signals that contribute to cellular senescence [[261], [262], [263], [264]]. Consequently, engineering biomaterials to present specific, pro-regenerative physical cues represents a promising approach for remodeling the senescent microenvironment.

Among these physical properties, substrate stiffness has emerged as a foundational factor. This is particularly true for mechanosensitive cells like macrophages, which are pivotal in orchestrating bone repair [265,266]. Recent studies have identified an optimal stiffness range where substrates with an intermediate elastic modulus can reprogram senescent macrophages (S-MΦs) from a M1 phenotype towards a M2 phenotype. For example, Bai et al. reported that a substrate stiffness of approximately 80 kPa mitigated inflammation and promoted bone regeneration in aged mice, a process linked to the inhibition of the mechanosensitive ion channel Piezo1 [267]. Exploring the downstream consequences, Zhang et al. found that S-MΦs cultured on 76 kPa hydrogels produced a conditioned medium that subsequently enhanced the angiogenic potential of senescent endothelial cells and the osteogenic differentiation of senescent BMSCs [268]. In contrast, substrates that were either substantially softer (∼18 kPa) or stiffer (∼295–300 kPa) failed to promote this beneficial M2 polarization. These findings reveal a multi-level mechanism where tuning substrate stiffness not only directly modulates senescent macrophages but also indirectly orchestrates a pro-regenerative microenvironment for bone repair.

**Fig. 9:**
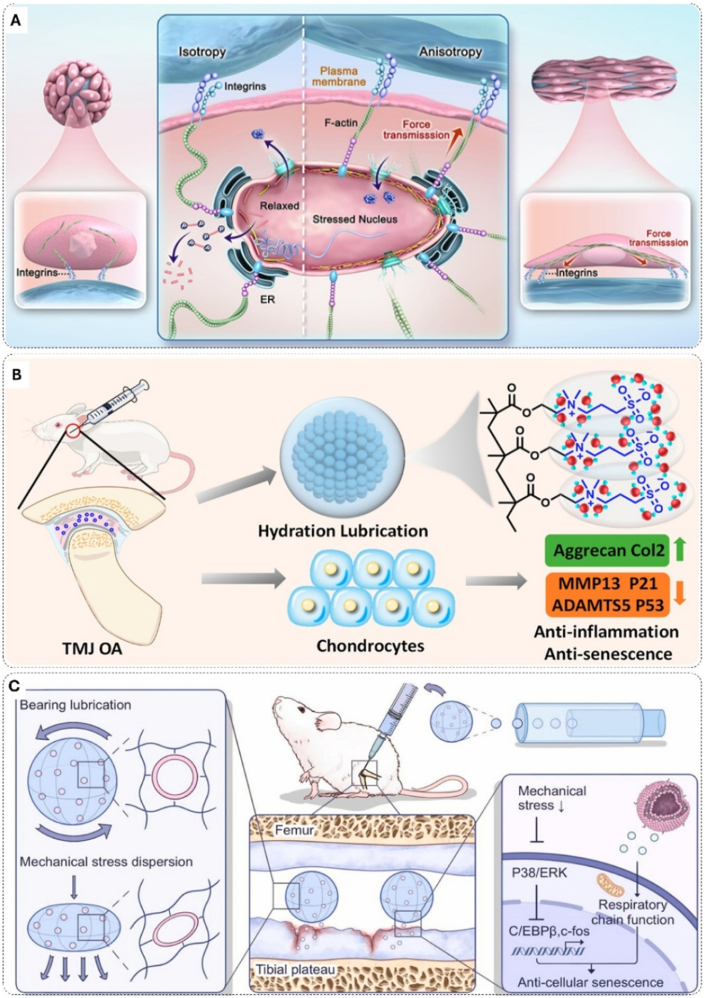
Biomaterial-based strategies for remodeling the senescent cell microenvironment through physical and mechanical modulation. A) MicroSphere 3D structures delay tissue senescence via mechanotransduction. Copyright 2025, American Chemical Society [271]. B) Bioinspired nanolubricant with anti-inflammatory and anti-senescence effects for treating TMJ osteoarthritis. Copyright 2022, American Chemical Society [273]. C) A hydrogel microsphere system (Res@Lipo@HMs) providing both mechanical stress dispersion and anti-senescence effects for OA therapy. Copyright 2025, Elsevier [274].

Shifting focus from the material's bulk properties to its surface features, engineering the surface topography at nano- and micro-scales offers another powerful dimension of physical control. The geometric landscape of a material's surface provides guidance that influences cell adhesion and morphology [269]. At the nanoscale, Sun et al. showed that arrays of titanium dioxide (TiO_2_) nanotubes with a 10 nm diameter could mitigate MSC senescence by activating the YAP signaling pathway, which promoted YAP's nuclear translocation and remodeled the distorted senescent nuclear envelope [270]. This principle extends to the microscale 3D geometry of scaffolds. Li et al. fabricated microgels as either anisotropic MicroRods or isotropic MicroSpheres, finding that the elongated shape of the MicroRods guided NP cells into a stretched morphology that also activated the YAP/TAZ pathway [271]. This mechanotransduction maintained nuclear membrane integrity by upregulating Lamin B1, thereby preventing nuclear DNA leakage and inhibiting the pro-inflammatory cGAS-STING signaling cascade (Fig. 9A). This resulted in an approximately threefold reduction in senescent cells compared to the MicroSphere group. These studies collectively demonstrate that enforcing a physiologically stretched cell morphology, via either nanoscale textures or microscale constraints, is a potent strategy to counteract senescence.

In contrast to the strategies of engineering static cues to actively guide cell behavior, a complementary approach aims to dynamically shield cells from deleterious external forces, which are primary drivers of senescence in mechanically active environments like joints [272]. This concept of mechanical shielding is achieved through the two primary mechanisms of biolubrication and stress dissipation. One approach focuses on biolubrication to reduce interfacial friction. Zhao et al. developed self-assembling, drug-free nanomicelles that function as effective lubricants via a hydration lubrication mechanism, reducing the coefficient of friction to as low as 0.022 [273]. This enhanced lubrication protected resident cells from friction-induced senescence and demonstrated a mechanoprotective principle with significant promise for bone regeneration within the challenging articular environment (Fig. 9B). Beyond friction, another advanced strategy aims to dissipate compressive loads. Departing from traditional high-stiffness models, Xu et al. developed highly deformable hydrogel microspheres that provide a microscopic cushioning effect [274]. By deforming under pressure to distribute load, they reduced high localized stress on individual chondrocytes, preserving mitochondrial function and decreasing stress-induced apoptosis (Fig. 9C). This concept of stress dispersion offers a novel paradigm for bone biomaterials, especially in load-bearing sites, by creating a more benign niche that minimizes a key extrinsic trigger of cellular senescence.

### 5.3 Remodeling via active signal delivery to reprogram biological communication

Beyond remodeling physical and chemical landscapes, effective intervention in the senescent microenvironment requires targeting its biological communication networks [275]. This milieu is characterized by aberrant intercellular signaling, where the SASP contributes to a dominant, pro-inflammatory network that tends to suppress tissue repair [276]. To counteract this, biomaterials are engineered as active platforms for signal delivery, designed to intercept pathological communication and initiate pro-regenerative dialogues. This biological reprogramming is achieved via two primary strategies that involve correcting the dysregulated immune microenvironment and modulating the endogenous stem cell pool.

#### 5.3.1 Shifting the immune microenvironment toward regeneration

Modulating the immune microenvironment is a central objective in reprogramming the biological communication networks of senescent tissues. In this state, resident immune cells, particularly macrophages, polarize towards a persistent M1 phenotype, and their secretion of SASP factors suppresses endogenous repair [277]. Consequently, a core therapeutic strategy is to induce the repolarization of these macrophages towards an M2 phenotype. Engineered biomaterials achieve this immunomodulation through distinct mechanisms, ranging from broad conditioning of the local environment to precise intervention in specific intracellular signaling pathways.

**Fig. 10:**
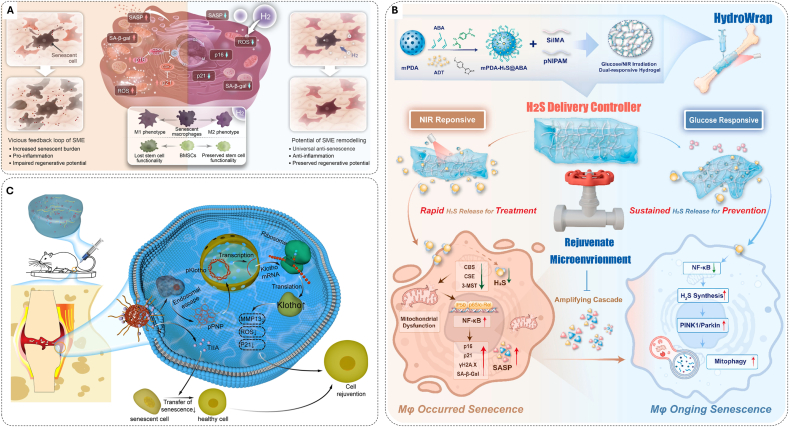
Therapeutic reprogramming of the SBM by modulating intercellular signaling pathways. A) Local H_2_ release remodels the senescent microenvironment in vitro, demonstrating anti-inflammatory and anti-senescence effects. Copyright 2023, The Author(s) [278]. B) Schematic of the HydroWrap H_2_S-releasing system designed to accelerate diabetic fracture healing by disrupting the senescence cascade. Copyright 2025, KeAi Communications Co., Ltd [279]. C) A pPNP-gel that generates Klotho protein to delay arthritis progression by improving the senescent microenvironment. Copyright 2024, Springer Nature [282].

One approach involves altering the extracellular milieu through the controlled release of therapeutic gasotransmitters, which temper the local oxidative and inflammatory state, thereby directly influencing macrophage polarization. Biomaterial platforms are critical for this strategy, as they enable localized and sustained gas delivery, overcoming the limitations of poor solubility and rapid diffusion associated with traditional administration. For instance, a scaffold developed by Chen et al. provided sustained H_2_ release for up to one week from encapsulated CaSi_2_ nanoparticles [278]. This localized H_2_ supply exerted a pervasive anti-senescence effect on multiple cell types and, crucially, repolarized senescent macrophages towards a regenerative M2 state (Fig. 10A). This shift was evidenced by a change in the M1/M2 macrophage ratio from a pro-inflammatory 1.91 to a pro-regenerative 0.71 in a senescent mouse model, thereby transforming the local microenvironment by reducing key SASP components. More advanced systems have been engineered to tailor gas release to specific pathological cues. The “HydroWrap” system designed by Zhang et al. represents such an intelligent, dual-responsive hydrogel for delivering H_2_S to the challenging microenvironment of T2DM-associated bone defects, where endogenous H_2_S levels are significantly reduced (Fig. 10B). This system operates in two distinct modes to address different stages of macrophage senescence. An acute, NIR-triggered high-dose H_2_S release first mitigates mitochondrial dysfunction and suppresses the SASP cascade, while a subsequent chronic, glucose-responsive release prevents senescence progression by promoting mitophagy. These examples illustrate how biomaterials delivering gasotransmitters can globally condition the microenvironment, with advancements enabling stimulus-responsive control for more dynamic immunomodulation [279].

A more refined strategy directly targets the intracellular signaling hubs that sustain the pro-inflammatory M1 phenotype. The cGAS-STING pathway, a critical driver of innate immunity and the SASP, serves as a prominent target. In the context of a high-ROS microenvironment modeling cartilage defects, He et al. reported a hydrogel containing Prussian blue nanozymes to counteract this pathway. Operating upstream, the nanozymes catalytically scavenge excess ROS. This action protects mitochondria from damage, thereby preventing the leakage of cytosolic mitochondrial DNA (mtDNA) that would otherwise activate the cGAS-STING cascade in macrophages. By intercepting this signaling cascade at its origin, the material effectively attenuated M1 polarization in bone marrow-derived macrophages and helped restore a pro-regenerative immune balance [280].

Building on this principle of precision, a further level of immunomodulatory control is achieved by targeting the subcellular source of inflammatory signaling itself, the mitochondrion. To address the pathological microenvironment of diabetic bone defects, Deng et al. developed a 3D-cryoprinted scaffold delivering a hybrid nanozyme for mitochondrial therapy. This nanozyme combines a ROS-scavenging manganese dioxide core with the SS31 peptide for mitochondrial targeting. By specifically eliminating mitochondrial ROS within macrophages, it suppressed the M1 phenotype and promoted M2 polarization, with the in vitro M2 population (F4/80^+^CD206^+^ cells) increasing from 51.89 % to 71.44 %. This approach remodels the immune environment for diabetic bone regeneration by intervening at the foundational level where aging-associated inflammation originates [281].

These studies demonstrate a spectrum of biomaterial-based strategies with increasing mechanistic precision. Whether through broad environmental conditioning via gasotransmitters, targeted inhibition of signaling hubs like cGAS-STING, or direct intervention at the mitochondrial source, all these approaches functionally modulate macrophages. By shifting the macrophage phenotype towards a pro-regenerative state, these materials dismantle the SASP-driven inflammatory niche and create a permissive microenvironment for subsequent tissue regeneration.

#### 5.3.2 Reestablishing pro-regenerative communication networks

Effective tissue repair ultimately depends on the recruitment and coordinated function of endogenous stem cells. While immunomodulation creates a more permissive niche, a complementary strategy involves directly engaging this core regenerative machinery. The senescent microenvironment presents a dual challenge by diminishing the pool of functional stem cells and dysregulating the signaling networks that guide their activity. Advanced biomaterials are engineered to overcome these deficits, first by recruiting regenerative cells and then by reprogramming local biological communication to restore their pro-regenerative capacity.

One strategy employs multi-functional platforms that deliver a synergistic combination of bioactive molecules to simultaneously address multiple barriers. An illustrative example is a stem cell-homing hydrogel developed by Wang et al. for osteoarthritis treatment [282]. This system integrates three distinct actions. It first recruits endogenous mesenchymal stem cells using the homing peptide PFSSTKT. Concurrently, it interrupts pathological communication by delivering Tanshinone IIA to inhibit Cx43 and CCN1 expression. Finally, it promotes pro-regenerative signaling through the delivery of Klotho-encoding plasmid DNA, which induces local cells to produce therapeutic factors (Fig. 10C). This integrated approach successfully reversed the inhibitory microenvironment, reducing the OARSI score in a rat model from 5 in controls to approximately 1 after 10 weeks.

A more sophisticated approach introduces sequential, process-oriented control, transforming the biomaterial into a dynamic platform that executes programmed tasks. Lei et al. demonstrated this recruit-and-rejuvenate strategy with a functionalized 3D-printed Ti6Al4V implant for osteoporotic bone repair [283]. The intervention proceeds in a defined sequence. The process begins as E7 peptides immobilized on the implant surface selectively capture endogenous bone marrow stromal cells. Following this capture, co-immobilized exosomes act upon the anchored cells to reverse their senescent phenotype and restore osteogenic potential. This sequential design elevates the biomaterial from a static scaffold to a dynamic platform, increasing the new bone volume fraction within the implant from 10 % in controls to nearly 30 % after 3 months in an osteoporotic rat model.

These advances mark a conceptual shift, redefining biomaterials as active therapeutic platforms capable of coordinating complex biological interventions within the senescent microenvironment. The integration of immunomodulation with targeted cell recruitment and signaling pathway regulation provides a comprehensive framework for addressing age-related tissue decline. Optimizing the spatiotemporal coordination of these functions represents a critical next step. Success in this area holds the potential to translate these active platforms into robust clinical therapies that can reliably promote regeneration in the non-permissive aged niche where conventional implants often fail.

## 6 The translational pathway for senescence-targeting bone biomaterials

The development of senescence-targeting bone biomaterials offers a promising solution to the clinical shortcomings of bone regeneration in the elderly. Extensive clinical use has established that conventional bone substitutes primarily function as passive structural supports [284,285]. This approach is insufficient to overcome the biological barriers imposed by an aged tissue niche. The translation of next-generation materials from a preclinical proof-of-concept to a clinical reality is therefore a paramount objective. This transition from passive scaffolding to active biological intervention necessitates navigating a distinct and formidable landscape of scientific, manufacturing, and regulatory challenges.

The primary scientific challenges are rooted in therapeutic precision and the predictive value of preclinical models. A fundamental biological hurdle is the heterogeneity of senescent cells, which can exhibit both detrimental and reparative phenotypes [147,286]. Indiscriminate cellular elimination risks ablating populations required for normal tissue repair, a concern highlighted by first-generation senolytics like Navitoclax, whose clinical use has been limited by off-target toxicities [287,288]. Even with more precise strategies like CAR-T cell therapies [289], significant safety and control issues persist [290,291]. This targeting dilemma is amplified within the aged host, where chronic inflammation can alter material degradation kinetics and disrupt the intended therapeutic window [292,293], a performance gap exemplified by in vivo data showing a threefold reduction in bone formation in aged mice compared to young controls using the same biomaterial [4].

These biological complexities are compounded by the limitations of established preclinical models. Foundational research often relies on rodent models, whose skeletal biology lacks the Haversian remodeling systems of humans [294]. Critically, these models often fail to fully recapitulate the chronic, low-grade inflammatory microenvironment of the aging human skeleton, a key therapeutic target. The development of large animal models that better reflect human bone physiology may therefore be essential for improving translational success [295,296]. The history of BMPs, where extensive animal testing did not adequately predict clinical side effects, serves as a pertinent cautionary tale for any potent, biologically active therapy [297,298]. Beyond the choice of animal model, establishing rigorous benchmarks for success is equally critical. To this end, future preclinical studies should more routinely incorporate healthy, young control groups to evaluate true functional restoration as opposed to merely partial repair.

Beyond these preclinical barriers, significant manufacturing and regulatory hurdles remain. The consistent, large-scale production of multi-component biomaterials that deliver therapeutic agents with controlled release profiles under Good Manufacturing Practices is a primary industrial challenge [[299], [300], [301]]. From a regulatory perspective, a core obstacle is the lack of a defined approval pathway for therapies targeting fundamental aging processes [302]. This ambiguity forces such innovations into ill-suited, disease-specific frameworks and complicates their classification as a drug, a device, or a combination product [303]. The absence of validated biomarkers to quantify senescent cell burden in humans further impedes the demonstration of therapeutic efficacy in clinical trials [304].

Addressing these convergent challenges requires a synergistic approach. Central to this is the advancement of non-invasive biomarkers capable of quantifying therapeutic effects. Emerging techniques in liquid biopsy, such as the analysis of circulating senescence-associated molecules, alongside established medical imaging, hold significant potential for monitoring the in vivo response to these materials [[305], [306], [307], [308]]. In parallel, pioneering studies such as the Targeting Aging with Metformin trial are establishing crucial precedents for geroscience-based regulatory endpoints [309,310]. The successful translation of senescence-targeting bone biomaterials will ultimately depend on the integration of these diagnostic and regulatory advances with rational material design, paving a viable path from laboratory concept to a clinical solution for regenerating the aging skeleton.

## 7 Future perspectives

**Fig. 11:**
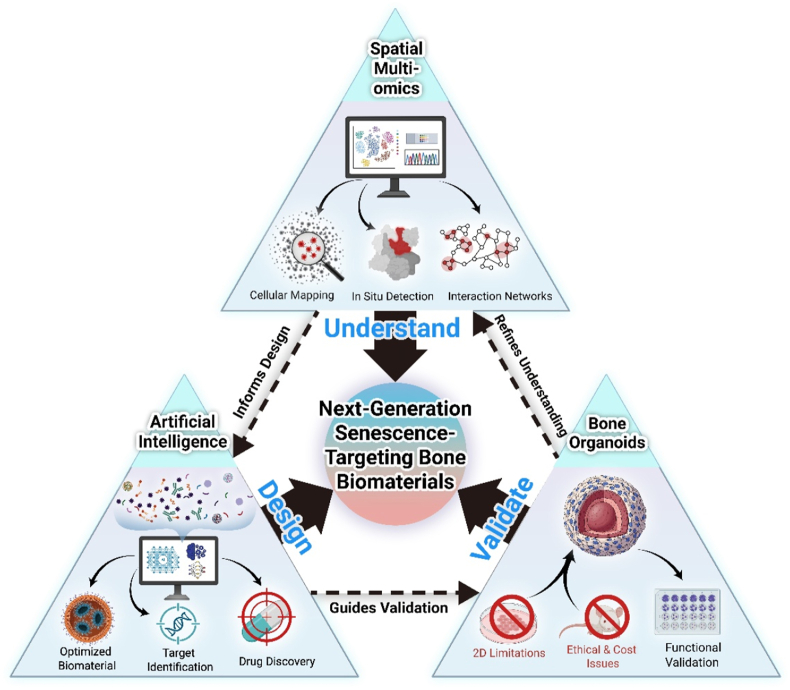
An integrated framework for developing senescence-targeting bone biomaterials. This framework integrates three technologies: spatial multi-omics characterizes the biological environment, AI guides material design, and bone organoids used for high-fidelity Validation. These components work iteratively (dashed arrows), where insights from one stage inform the next, creating a feedback cycle that accelerates the transition from empirical discovery to a more predictive science.

Targeting senescent cells offers potential for aged bone regeneration, though their heterogeneity and tissue-specific phenotypes present design challenges. This transformation could be catalyzed by the convergence of three synergistic fields. These include spatial multi-omics for deepening mechanistic understanding, AI for predictive design, and bone organoid models for high-fidelity validation [311]. Together, these pillars can form an integrated, closed-loop framework for developing next-generation biomaterials capable of navigating the complex landscape of cellular senescence within the SBM (Fig. 11).

### 7.1 Deepening mechanistic understanding through spatial multi-omics

A granular comprehension of the biomaterial-host interface, particularly the complex interactions involving senescent cells, is the foundation for targeted bone regeneration. Spatial omics technologies are facilitating a fundamental shift from a generalized view of this interface to a high-resolution map of specific cell subtypes, their precise spatial localization, and their communication networks within the bone regenerative niche [312,313]. The goal of applying these technologies is to construct a Biomaterial-mediated Cell Atlas (BCA), a framework designed to systematically chart the multi-omics responses of host cells, including senescent populations, to a biomaterial [314]. Such an approach moves beyond simple biocompatibility assessments to gain deep mechanistic insights into how a material modulates the local senescent microenvironment.

These detailed atlases are generated by integrating single-cell transcriptomics with spatially resolved technologies like MERFISH or Xenium, which allows for a comprehensive characterization of cellular diversity while preserving tissue architecture [315,316]. Analysis of a BCA can enable researchers to pinpoint specific surface markers on senescent cells for targeted biomaterial delivery or to identify key pathological signaling hubs within the regenerative niche as targets for therapeutic intervention [317]. The principle of this omics-guided design strategy was effectively demonstrated by Fan et al. [318]. In their work, single-cell transcriptomics was used to identify a pathological feedback loop driving skeletal stem cell senescence in aged bone. This mechanistic insight guided the rational application of RSV to disrupt the pathological cycle, with a smart hydrogel employed as the delivery vehicle to enhance osseointegration, offering a validated methodology for future omics-informed strategies.

Significant challenges, however, remain in applying these techniques to the complex interplay between biomaterials and senescent cells. The integration of multi-modal data is technically demanding and can be complicated by batch effects and analytical complexity [319]. Furthermore, the high cost and specialized expertise associated with these techniques currently limit their widespread accessibility [320]. Addressing these technical and economic barriers will be crucial for translating these powerful discovery tools into routine research and development pipelines.

### 7.2 Predictive design using AI

AI is positioned to transition biomaterial design from an empirical, trial-and-error process into a more predictive science [320]. This evolution holds particular promise for the development of biomaterials targeting senescent cells for bone regeneration, with applications unfolding across a spectrum from high-throughput screening to the autonomous discovery of novel materials.

At a foundational level, machine learning excels at identifying promising candidates from vast chemical libraries and predicting material performance [321]. This capability has been demonstrated in bone-related research where models have predicted the osteoinductive properties of biomaterials with high accuracy (AUC of 0.921), thereby automating a critical screening process [322]. A compelling case study by Smer-Barreto et al. further highlights this potential; their machine learning model, trained on public data, identified three novel senolytic compounds from a library of over 4,000, reducing screening costs by several hundredfold [323].

Beyond screening existing candidates, a more advanced application involves the de novo design of materials with precisely tailored functions. This concept of AI-guided generative design is exemplified by the development of functional peptide hydrogels, where a computational platform was used to create a novel antimicrobial peptide and formulate it into a hydrogel system [324]. Such approaches enable the direct creation of biomaterials, like a biodegradable polymer with optimized release kinetics for a specific senolytic drug, engineered from first principles.

The ultimate manifestation of this data-driven methodology is the autonomous platform, or “self-driving lab,” which integrates machine learning algorithms with robotic experimentation to create a closed-loop discovery cycle [325,326]. High-profile examples such as A-Lab and Coscientist have demonstrated the capacity to autonomously synthesize and test novel compounds, signaling a new era in materials science [327,328]. Applying this concept to our field could dramatically accelerate the development of biomaterials that modulate the senescent cells.

However, two primary obstacles must be addressed to fully realize this potential. First, the “black box” nature of many complex AI models presents a significant hurdle for clinical translation, where mechanistic understanding and model transparency are critical for regulatory approval and risk assessment [[329], [330], [331]]. Second, the performance of any AI model is contingent upon the quality and quantity of its training data. The current lack of standardized, high-quality datasets on biomaterial-senescent cell interactions remains a major bottleneck that will require a concerted community effort to resolve.

### 7.3 High-fidelity validation with bone and cartilage organoids

Bone and cartilage organoids are emerging as a critical validation platform, bridging the gap between simplistic 2D cell cultures and complex animal models by offering a human-relevant physiological context [332,333]. The field is rapidly advancing beyond basic structures to create functional and pathological models that can recapitulate specific aspects of disease [334]. A key example is the work by Boone et al., who established reliable, high-throughput cartilage organoid models of senescence using tissues from aged OA patients [335]. By applying controlled stimuli such as hyperphysiological mechanical loading or irradiation, they could induce distinct senescence profiles, including SASP-dominant or cell-cycle-arrest-dominant phenotypes [336,337]. This level of control transforms organoids into an invaluable platform for screening anti-senescence compounds in a multi-well format, providing a technical blueprint for developing analogous bone organoid models to directly evaluate senescence-targeting biomaterials [338].

Beyond their role as validation tools, organoid systems are evolving into therapeutic platforms themselves. The work by Sun et al. exemplifies this transition, demonstrating a composite hydrogel that delivers an anti-senescence microRNA (miR-24) via synovial mesenchymal stromal cell (SMSC) organoids [339]. This integrated system not only repaired cartilage defects in an osteoarthritic rat model but also alleviated the local senescent microenvironment, highlighting a potent synergy between biomaterials, organoids, and senescence-targeting therapeutics.

Achieving sufficient biological fidelity, however, remains a primary challenge for the field. Current models often lack mature vascularization and integrated immune components [340,341], elements known to critically modulate the SASP and the clearance of senescent cells [342]. As highlighted in recent analyses, advancing toward next-generation, applied organoid systems will likely require the precise spatial and temporal coordination of multiple tissue types, a significant ongoing engineering hurdle [340].

### 7.4 Concluding remarks and future outlook

The convergence of spatial omics, artificial intelligence, and organoid models suggests a future defined by a closed-loop, iterative discovery cycle [343]. In this cycle, deep biological insights from omics would inform the AI-driven design of novel biomaterials by self-driving labs [344]. These materials could then be rapidly validated and refined in patient-derived organoids that model senescence. Such an integrated approach has the potential to transform the development of senescence-targeting biomaterials from a largely empirical endeavor into a more predictive science. However, translating this powerful framework from concept to clinical reality will introduce substantial ethical and regulatory considerations. Issues of data privacy, biospecimen governance, and the safety of AI-designed materials and living therapeutics will demand proactive engagement from the scientific and clinical communities to ensure that such transformative innovation proceeds responsibly.

## CRediT authorship contribution statement

**Haitong Wu:** Writing – review & editing, Writing – original draft, Visualization. **Qing Zhang:** Writing – review & editing, Visualization, Conceptualization. **Jinhao Zhu:** Writing – original draft. **Lihong Wu:** Writing – review & editing, Supervision. **Yin Xiao:** Writing – review & editing, Supervision, Conceptualization. **Xuechao Yang:** Writing – review & editing, Supervision, Funding acquisition, Conceptualization.

## Ethics approval and consent to participate

Not applicable for this manuscript. This study did not involve animal/human participants, animal/human data, or animal/human tissue.

## Funding

This work was supported by the 10.13039/501100001809National Natural Science Foundation of China (32301129), 10.13039/501100003453Natural Science Foundation of Guangdong Province (No. 2023A1515012777), the Science and Technology Planning Project of Guangzhou (No. 202201020203, 202201020117), Featured Clinical Technique of Guangzhou (No. 2023C-TS58).

## Declaration of competing interest

The authors declare that they have no known competing financial interests or personal relationships that could have appeared to influence the work reported in this paper.

## Abbreviations

SASP: Senescence-associated secretory phenotype
BMSCs: Bone marrow mesenchymal stem cells
NF-κB: Nuclear factor kappa-B
BCL-2: B-cell lymphoma 2
SA-β-gal: Senescence-associated β-galactosidase
EVs: Extracellular vesicles
DDR: DNA damage response
cGAS-STING: Cyclic GMP-AMP synthase-Stimulator of interferon genes
mTOR: mammalian target of rapamycin
CFU-F: Colony-forming unit-fibroblast
PPARγ: Peroxisome proliferator-activated receptor gamma
RUNX2: Runt-related transcription factor 2
RANKL: Receptor activator of nuclear factor kappa-B ligand
OPG: Osteoprotegerin
SIRTs: Sirtuins
AMPK: AMP-activated protein kinase
γH2AX: gamma H2A histone family member X
Cx43: Connexin 43
SOST: Sclerostin
GATA4: GATA binding protein 4
CTSK: Cathepsin K
CDT: Cytolethal distending toxin
KLRG1: Killer cell lectin-like receptor G1
mTORC1: mammalian target of rapamycin complex 1
GCA: Grancalcin
BMAds: Bone marrow adipocytes
JAKi: JAK inhibitor
BMD: Bone mineral density
INK family: Inhibitor of cyclin-dependent kinase family
SBM: Senescent Bone Microenvironment
AGEs: Advanced glycation end products
D + Q: Dasatinib and Quercetin
MMPs: Matrix metalloproteinases
NP: Nucleus pulposus
IVDD: Intervertebral disc degeneration
Aln-Lipo-DQ: Alendronate-functionalized liposomes co-loaded with dasatinib and quercetin
BV/TV: Bone volume/total volume
FLS: Fibroblast-like synoviocytes
CX3-LS-DQ: CX3 aptamer-functionalized liposomes loaded with dasatinib and quercetin
OA: Osteoarthritis
OARSI: Osteoarthritis Research Society International
DM1-Gal: Galactose-modified maytansinoid prodrug
NK cells: Natural Killer cells
BGs: Bioactive glass nanoparticles
PCL: Polycaprolactone
PDA: Polydopamine
SrCS: Strontium chondroitin sulfate
RSV: Resveratrol
CeO_2_: Cerium oxide
NAC: N-acetylcysteine
T2DM: Type 2 diabetes mellitus
NR: Nicotinamide riboside
TPG: Tea polyphenol-reduced graphene
BBR: Berberine
TNTs: Tunneling nanotubes
iMVs: microvesicles from IFN-γ-prestimulated mesenchymal stem cells
EM-eNM: Energy metabolism-engaged nanomedicine
polyP: inorganic polyphosphate
TDNs: DNA tetrahedra
NPPCs: Nucleus pulposus progenitor cells
LNPs: Lipid nanoparticles
circRNA: circular RNA
HMSNs: Hollow mesoporous silica nanoparticles
m7G: N7-methylguanosine
Akt: Akermanite
MBG: Mesoporous Bioactive Glass
oi-EVs: osteoinductive EVs
MB: Magnesium boride
BP@Mg: Magnesium-modified black phosphorus
Mg-Ce-MOF: Magnesium-cerium metal-organic framework
S-MΦs: Senescent macrophages
TiO_2_: Titanium dioxide
YAP/TAZ: Yes-associated protein/transcriptional coactivator with PDZ-binding motif
BCA: Biomaterial-mediated Cell Atlas
SMSC: Synovial mesenchymal stromal cell
