# Supplementary material for: Biomaterials targeting senescent cells for bone regeneration: State-of-the-art and future perspectives
Source: Bioact Mater. 2025 Sep 8;54:686–714. doi: 10.1016/j.bioactmat.2025.09.002 (PMC12454288; doi:10.1016/j.bioactmat.2025.09.002)

**Biomaterials targeting senescent cells for bone regeneration: State-of-the-art and future perspectives**

**Copyright Permissions**

**An anti-senescence hydrogel with pH- responsive drug release for mitigating intervertebral disc degeneration and low back pain**


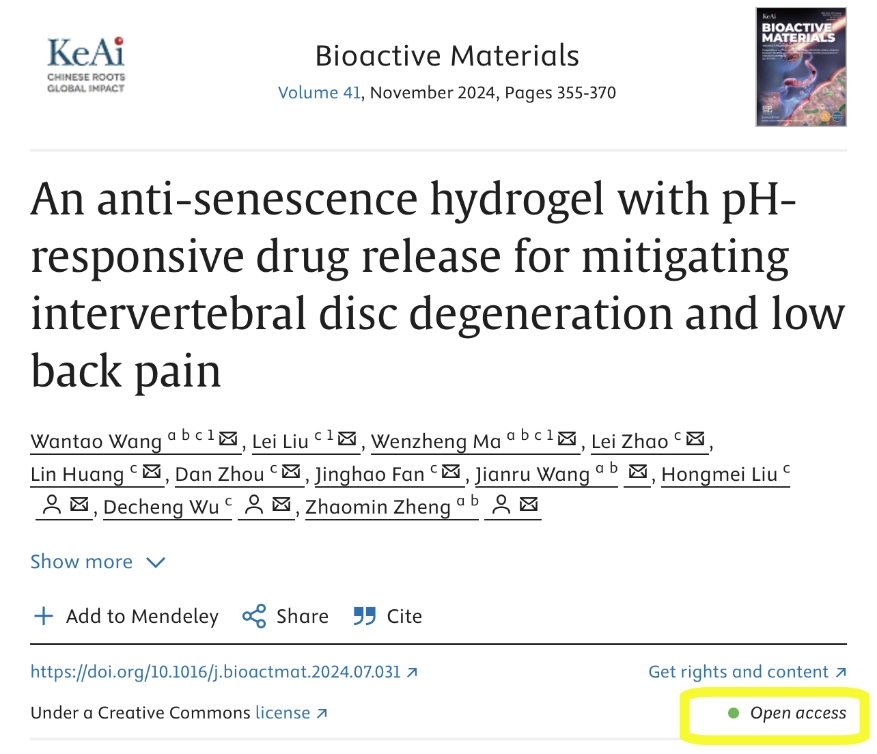


**Targeted delivery of liposomal senolytics to alleviate cellular senescence-induced bone loss**


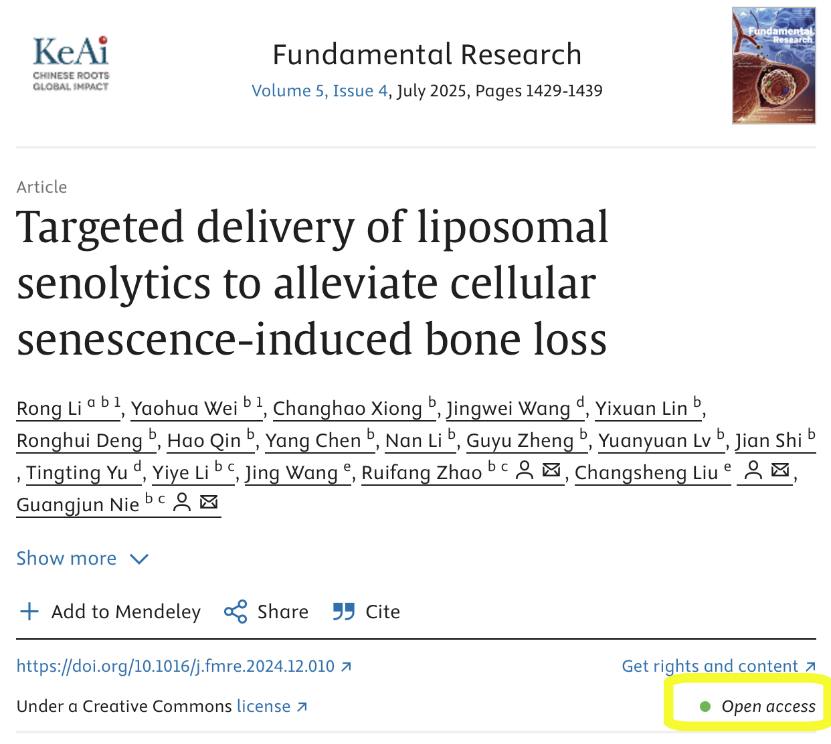


**Photoactivatable Exosenolytics Activate Natural Killer Cells for Delaying Osteoarthritis**


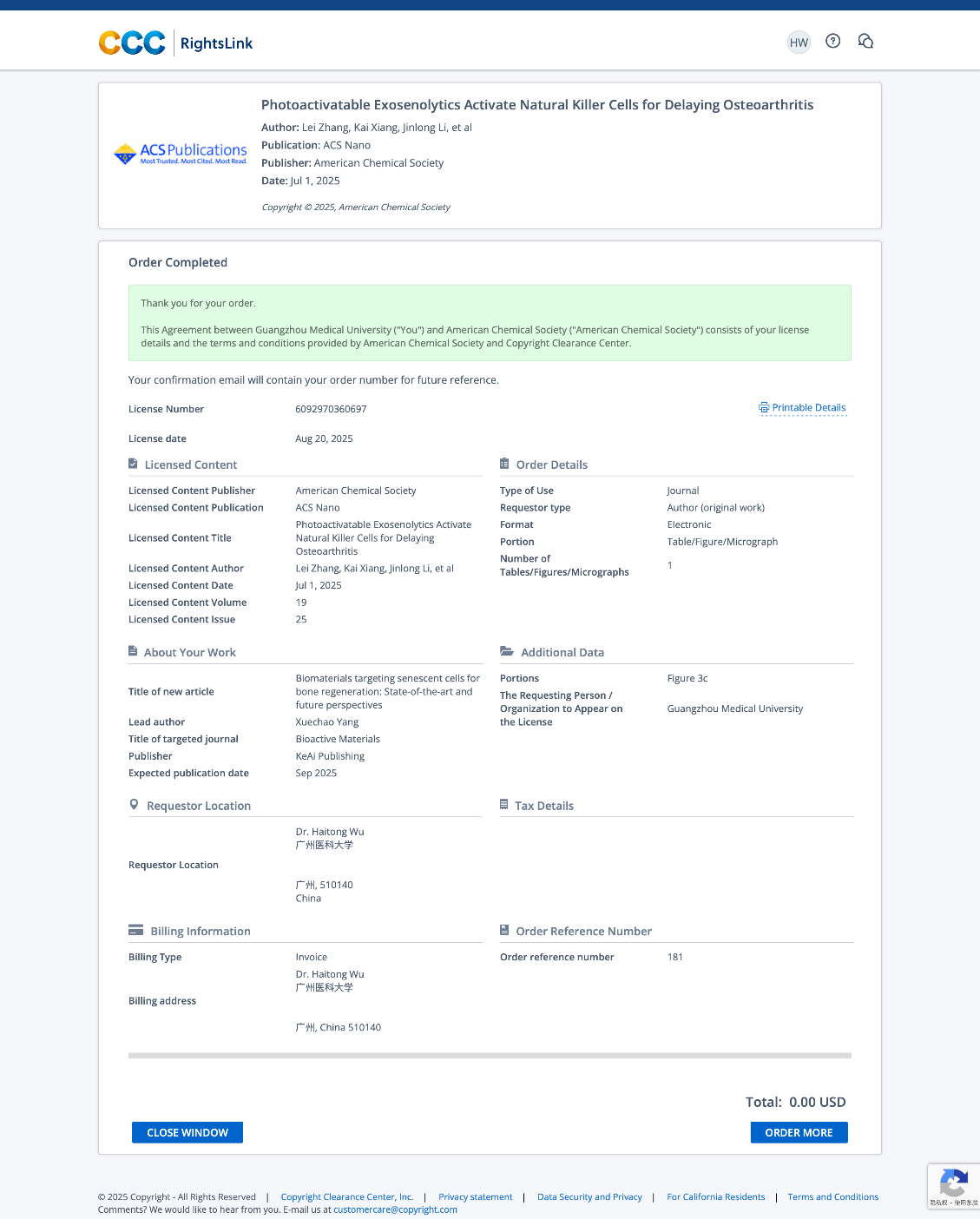


**Tissue Engineering of JAK Inhibitor-Loaded Hierarchically Biomimetic Nanostructural Scaffold Targeting**


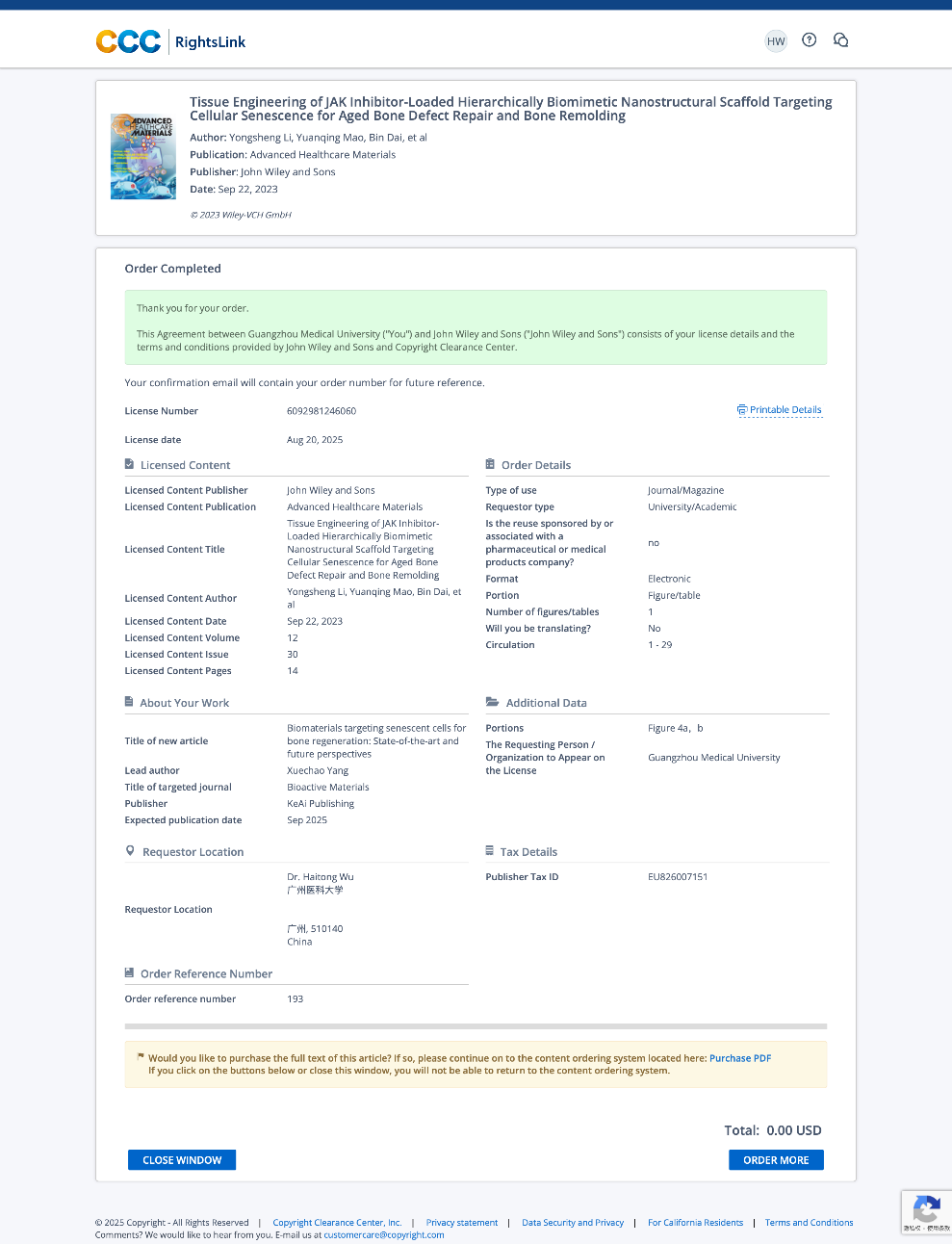


**Zwitterion-Lubricated Hydrogel Microspheres Encapsulated with Metformin Ameliorate Age-Associated Osteoarthritis**


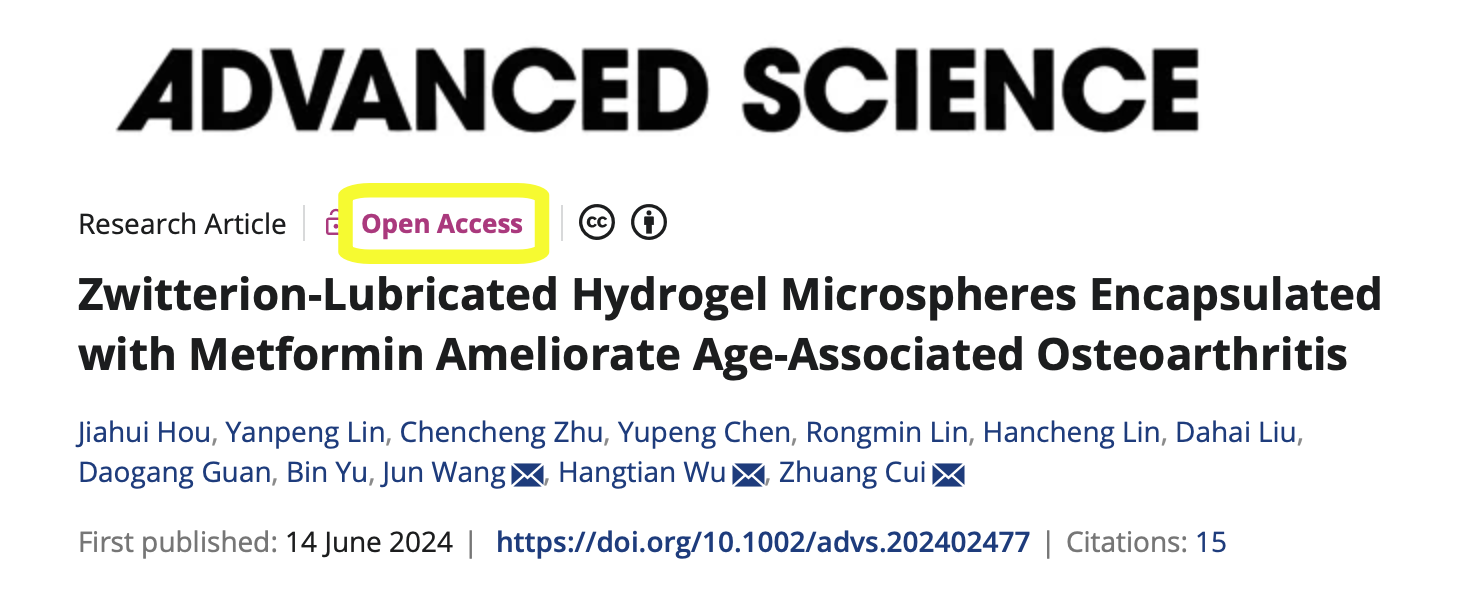


**Multifunctional CeO2 nanozymes for mitigating high-glucose induced senescence and enhancing bone regeneration in type 2 diabetes mellitus**


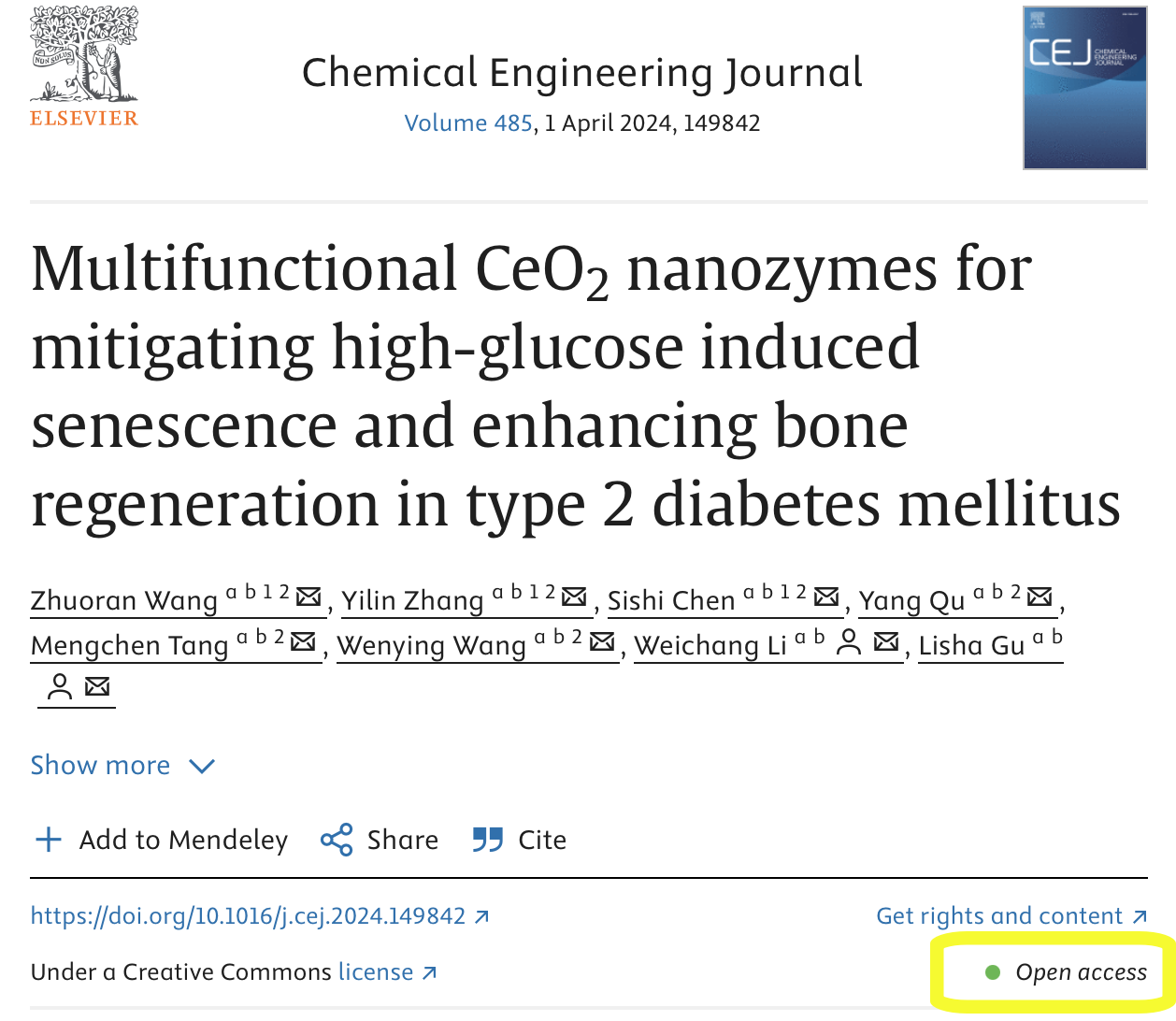


**Rejuvenating Aged Bone Repair through Multihierarchy Reactive Oxygen Species-Regulated Hydrogel**


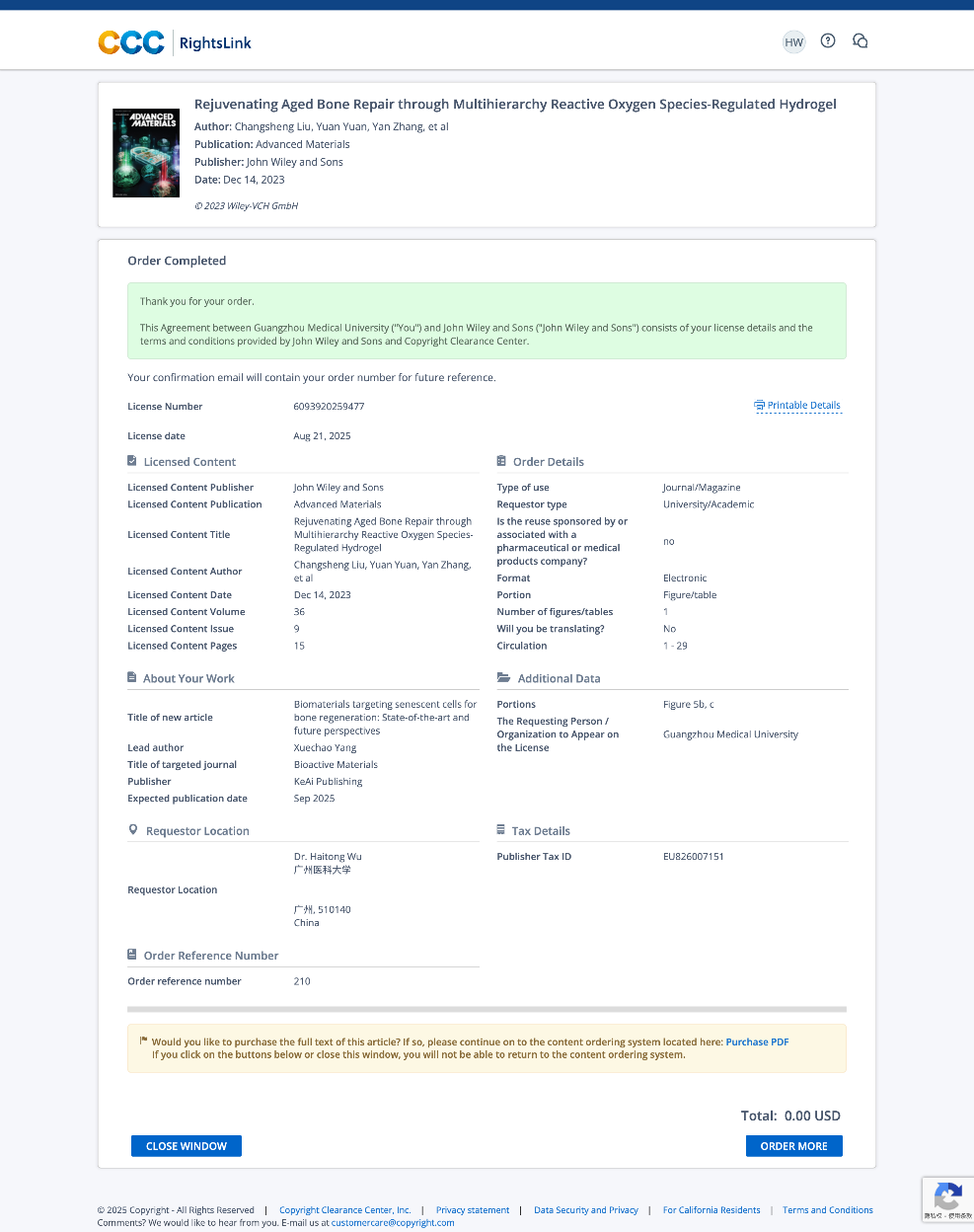


**Melatonin-loaded bioactive microspheres accelerate aged bone regeneration by formation of tunneling nanotubes to enhance mitochondrial transfer**


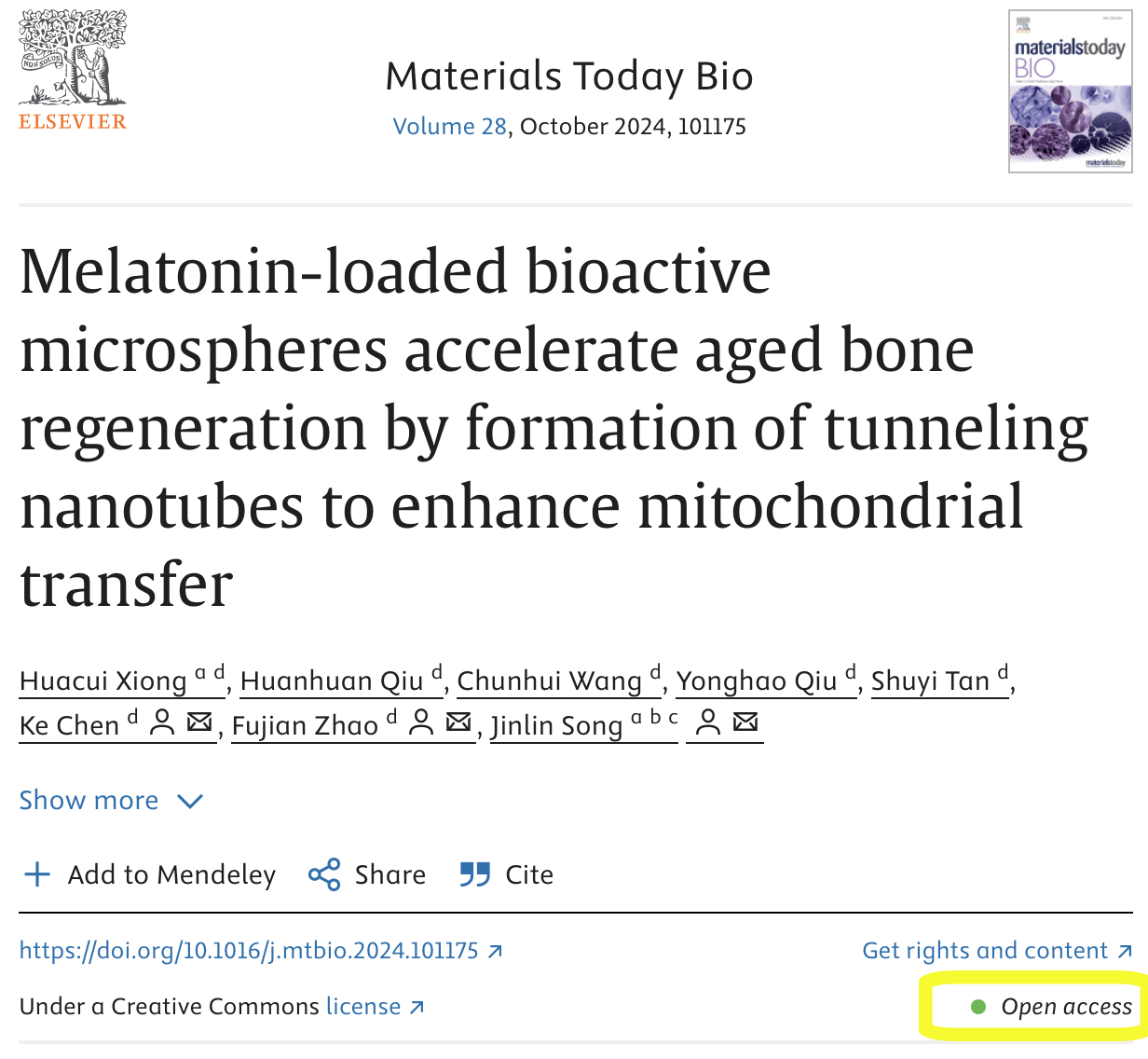


**Microvesicles-hydrogel breaks the cycle of cellular senescence by improving mitochondrial function to treat osteoarthritis**


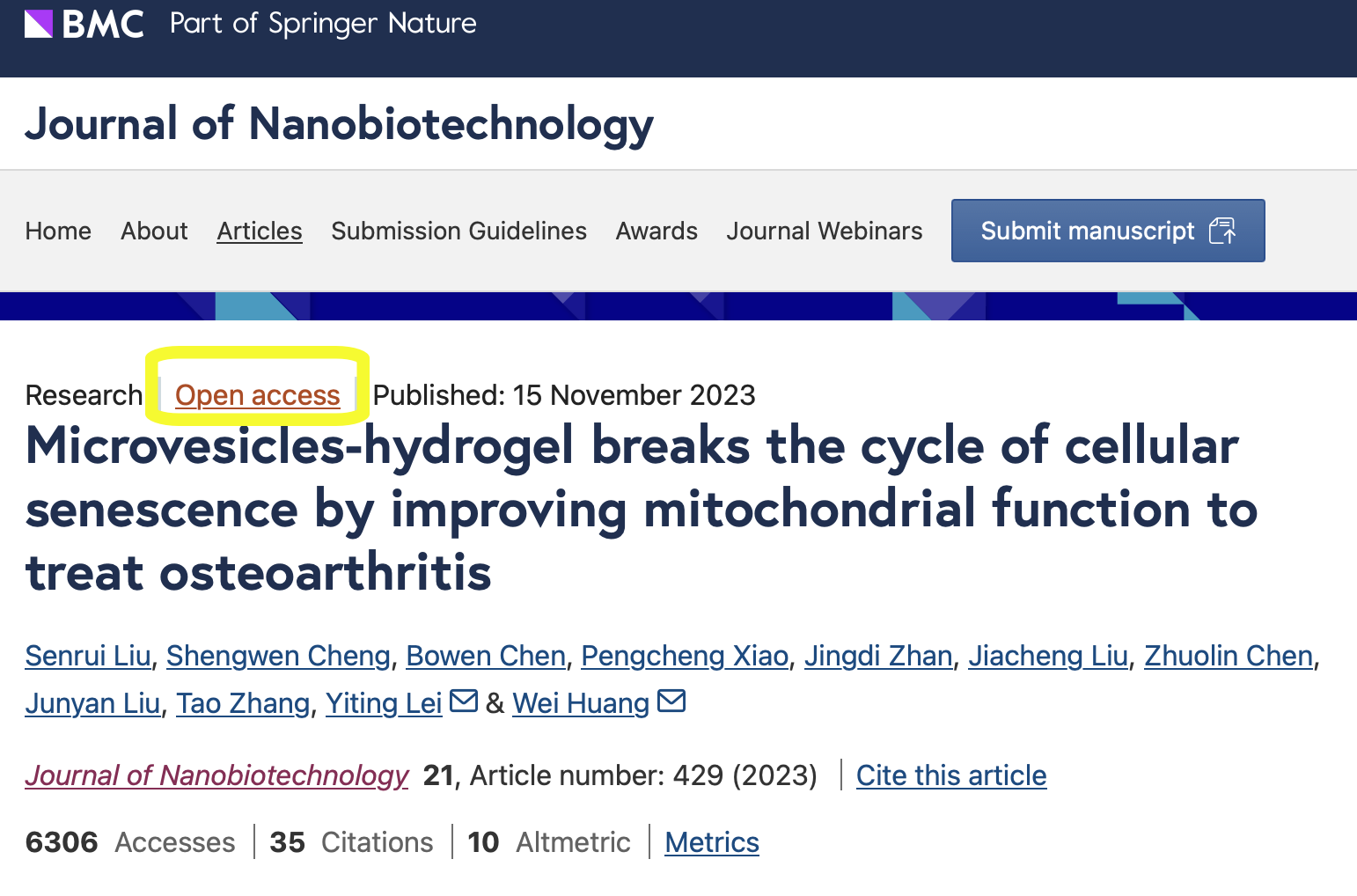


**DNA Tetrahedron Delivering miR‐21‐5p Promotes Senescent Bone Defects Repair through Synergistic Regulation of Osteogenesis and Angiogenesis**


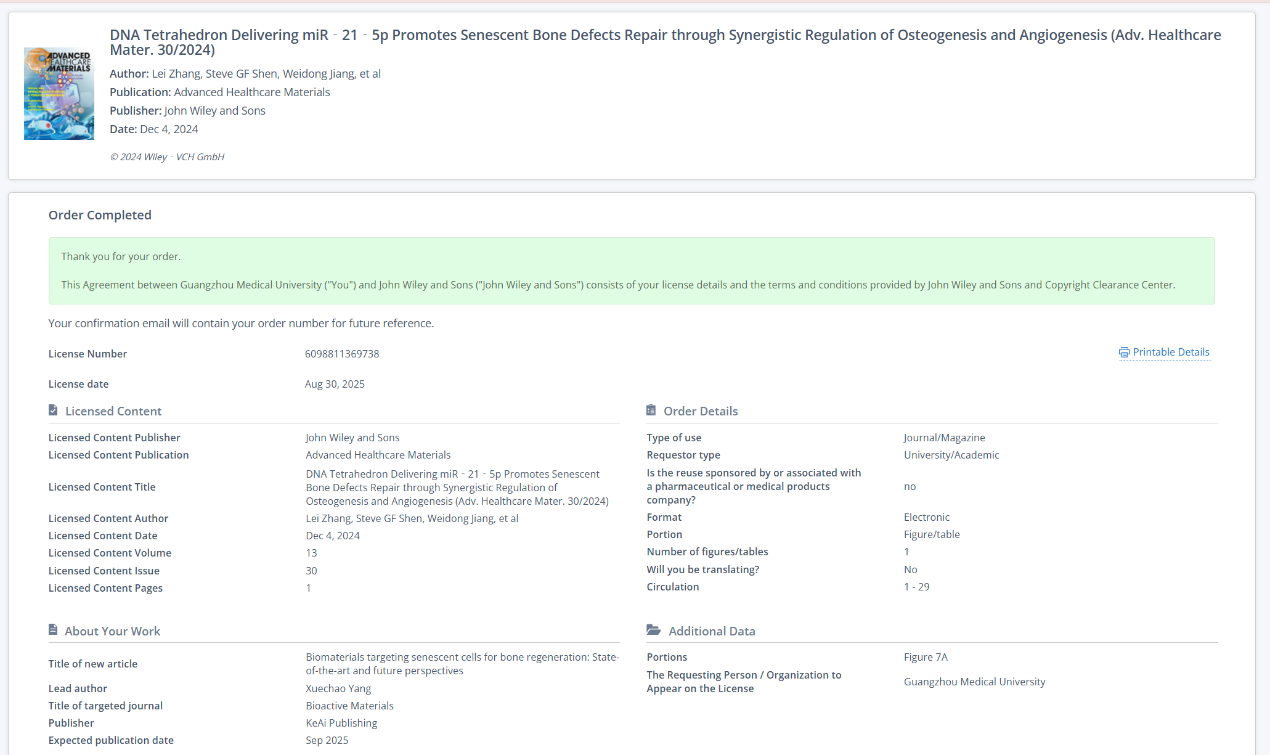


**Magnesium-containing bioceramics stimulate exosomal miR-196a-5p secretion to promote senescent osteogenesis through targeting Hoxa7/MAPK signaling axis**

**
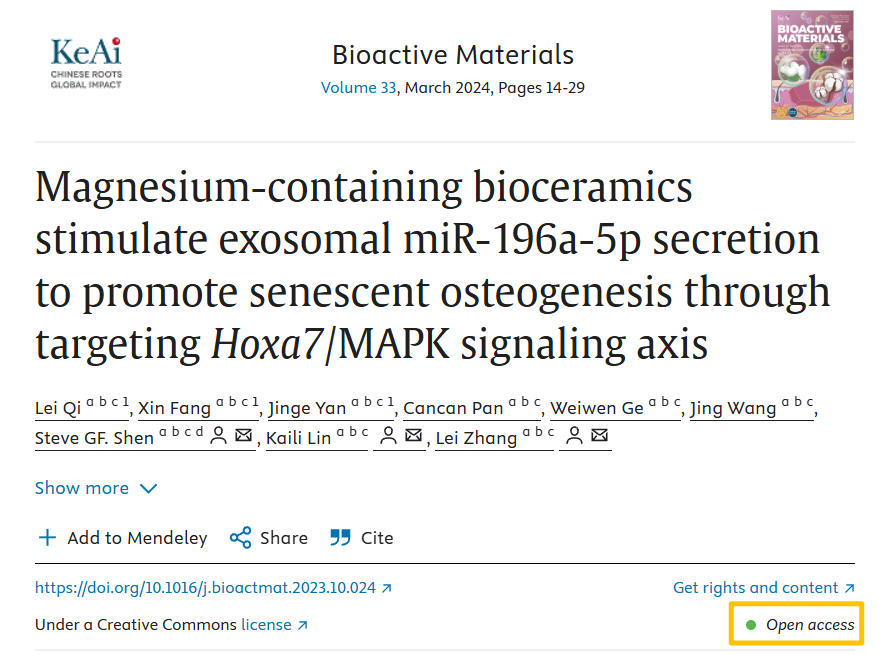
**

**Mesoporous bioactive glass scaffolds for the delivery of bone marrow stem cell-derived osteoinductive extracellular vesicles lncRNA promote senescent bone defect repair by targeting the miR-1843a-5p/Mob3a/YAP axis**


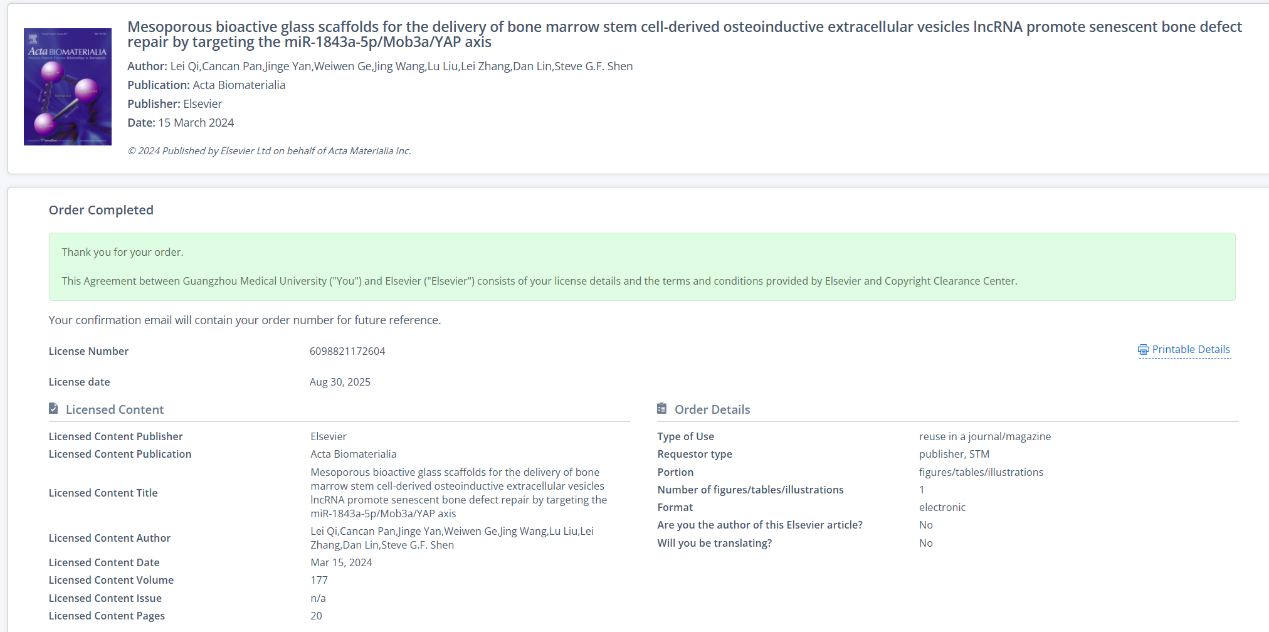


**Cell-Free Osteoarthritis Treatment with Sustained-Release of Chondrocyte-Targeting Exosomes from Umbilical Cord-Derived Mesenchymal Stem Cells to Rejuvenate Aging Chondrocytes**


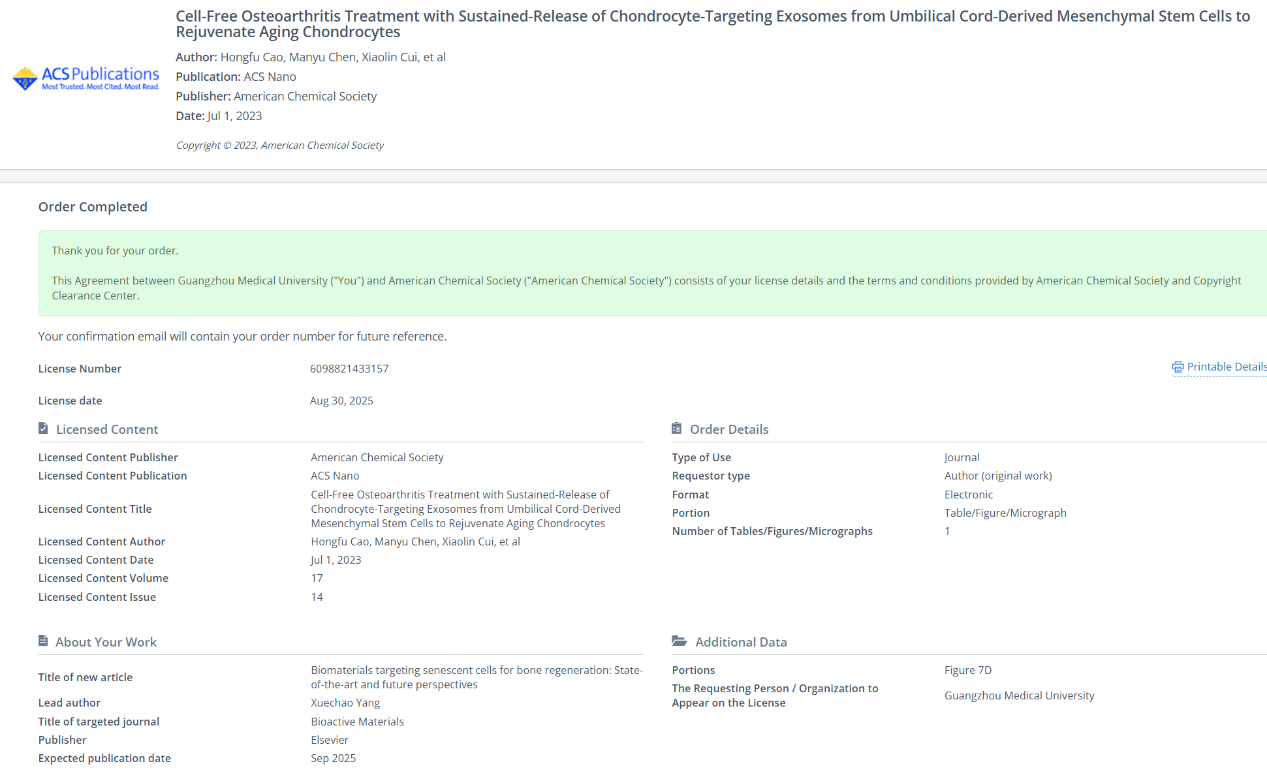


**Active Magnesium Boride/Alginate Hydrogels Rejuvenate Senescent Cells**


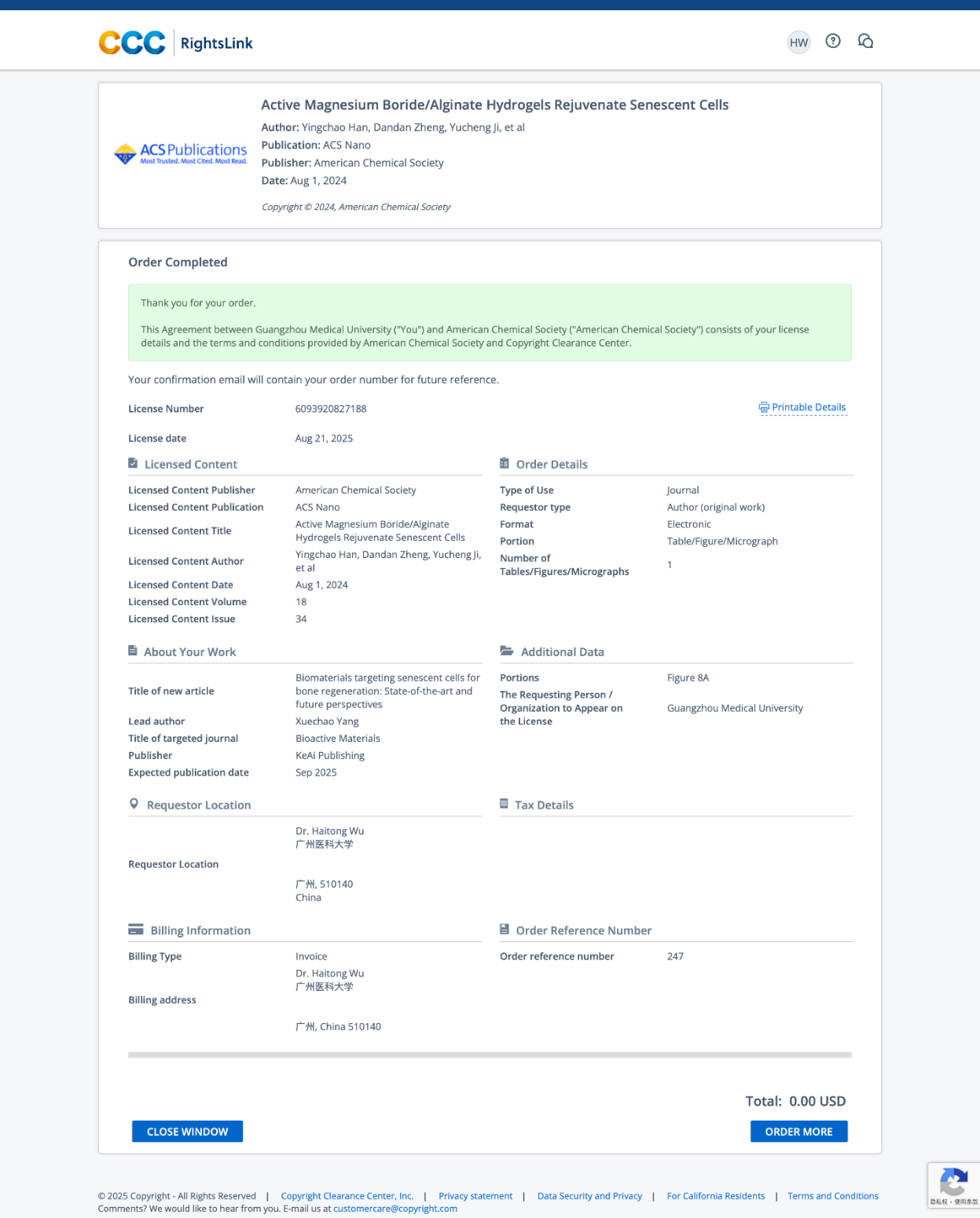


**An "inside-out"-guided genetically engineered hydrogel for augmenting aged bone regeneration**


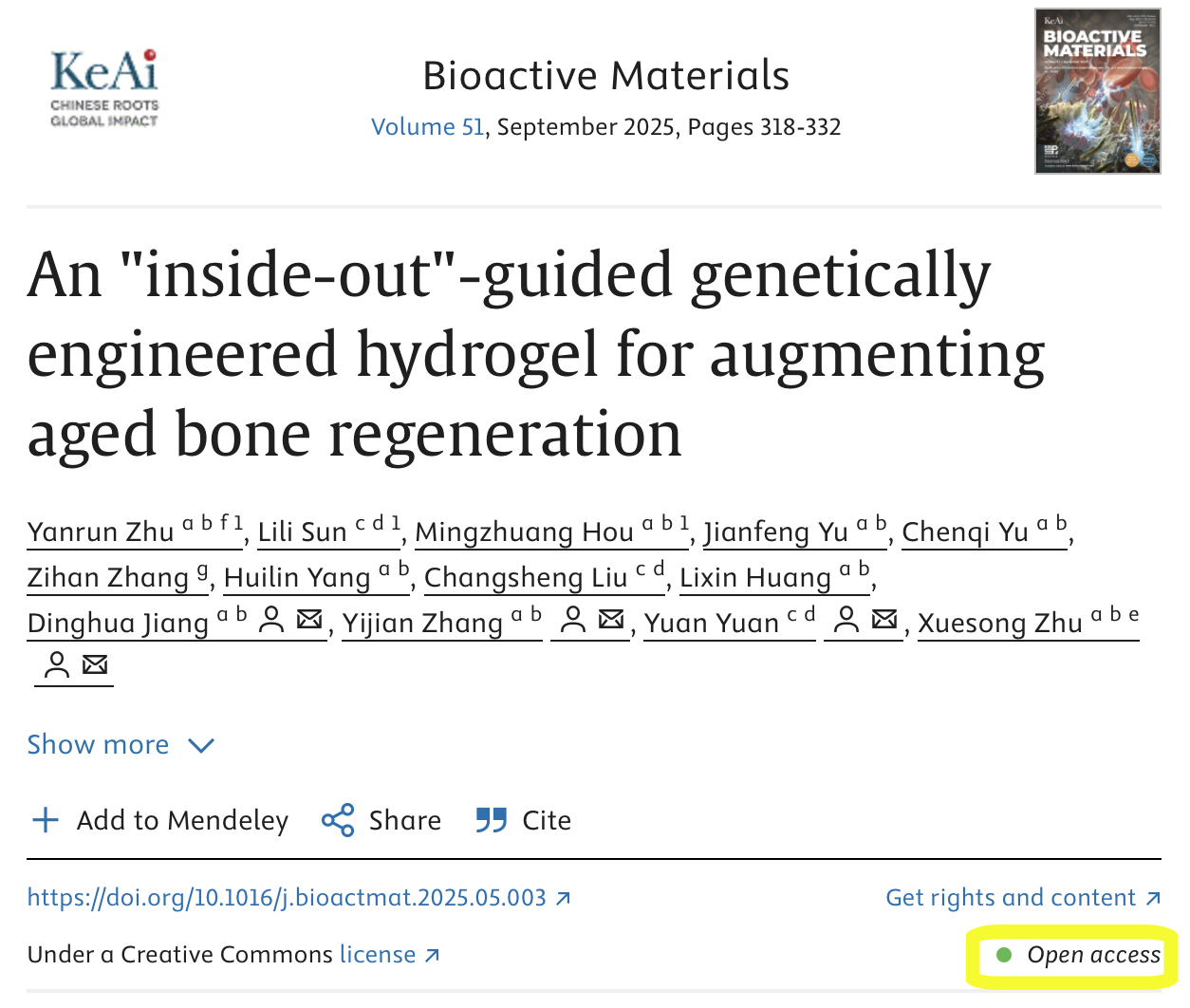


**MicroSphere 3D Structures Delay Tissue Senescence through Mechanotransduction**


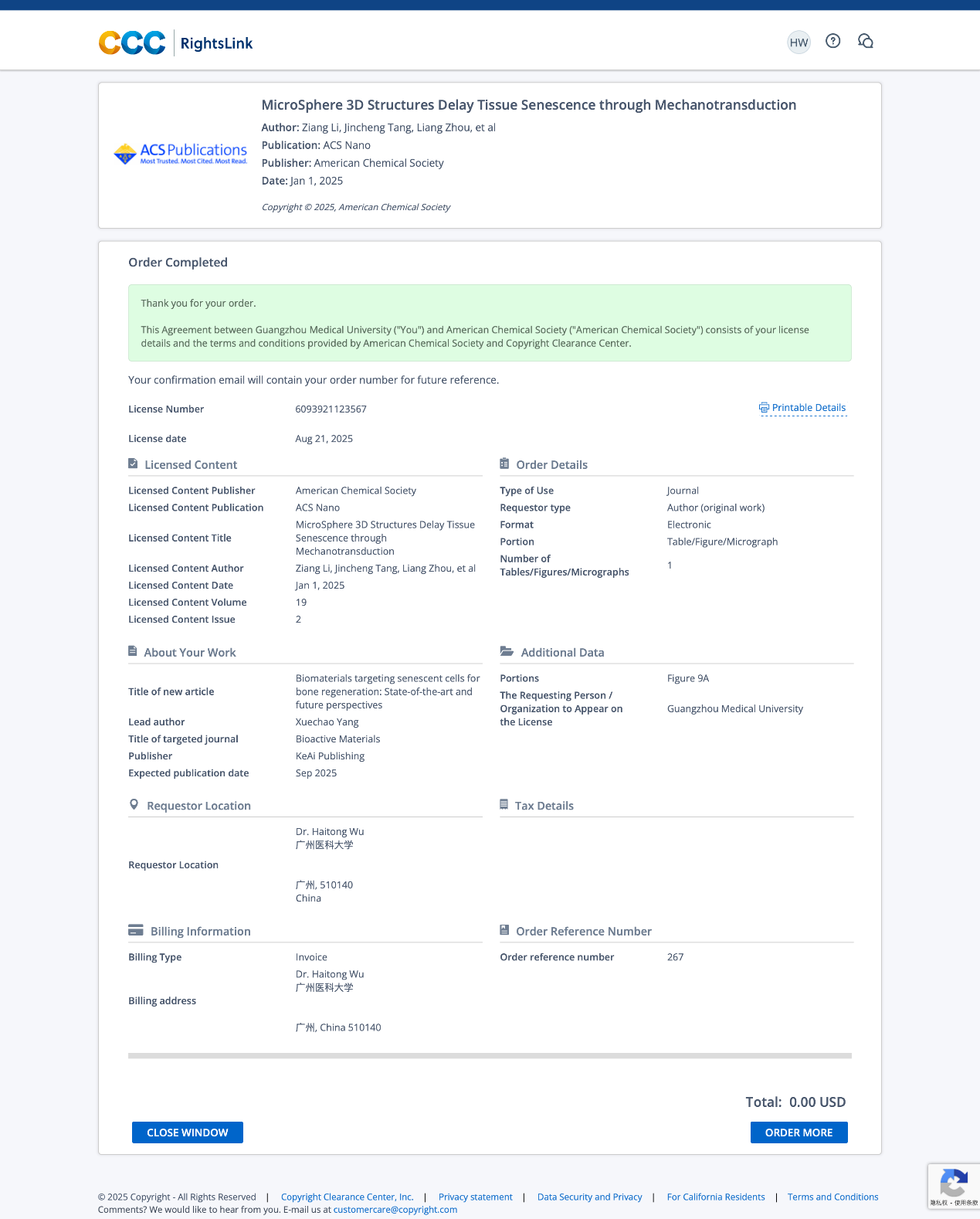


**Bioinspired Nanospheres as Anti-inflammation and Antisenescence Interfacial Biolubricant for Treating Temporomandibular Joint Osteoarthritis**


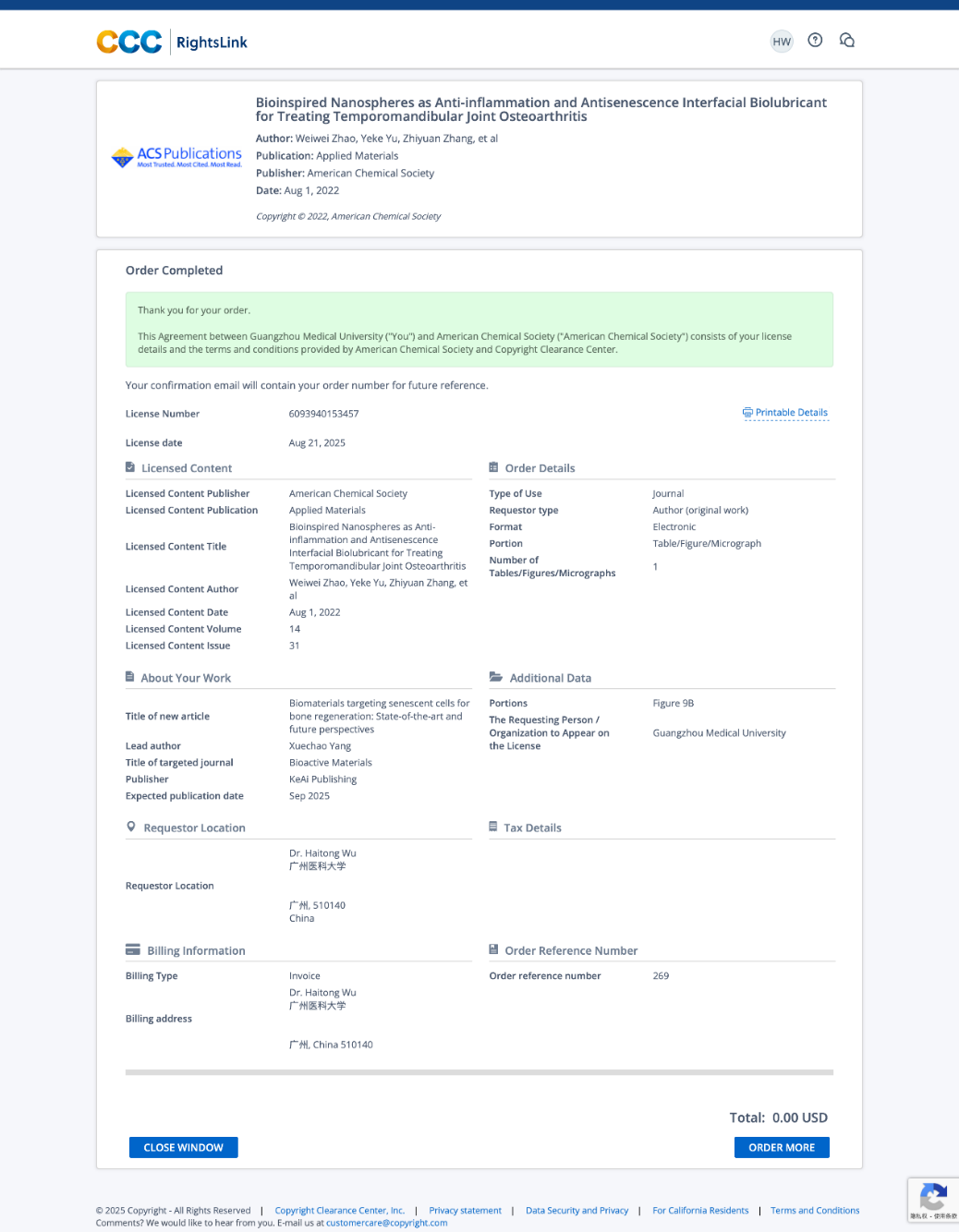


**High-mobility network hydrogel microsphere system to combat chondrocyte senescence for enhanced cartilage repair and regeneration**


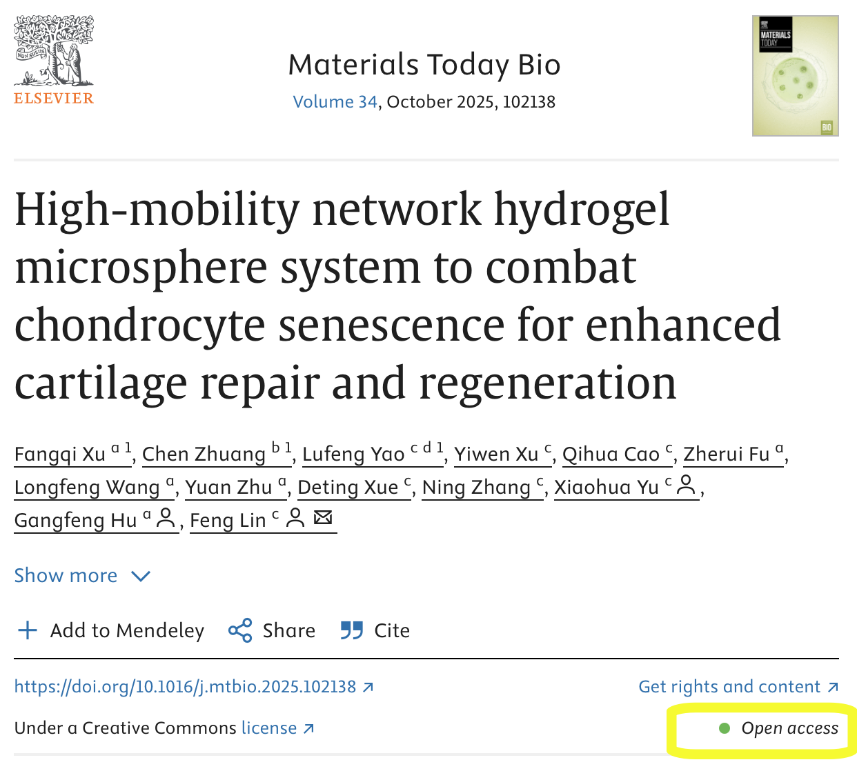


**Local H2 release remodels senescence microenvironment for improved repair of injured bone**


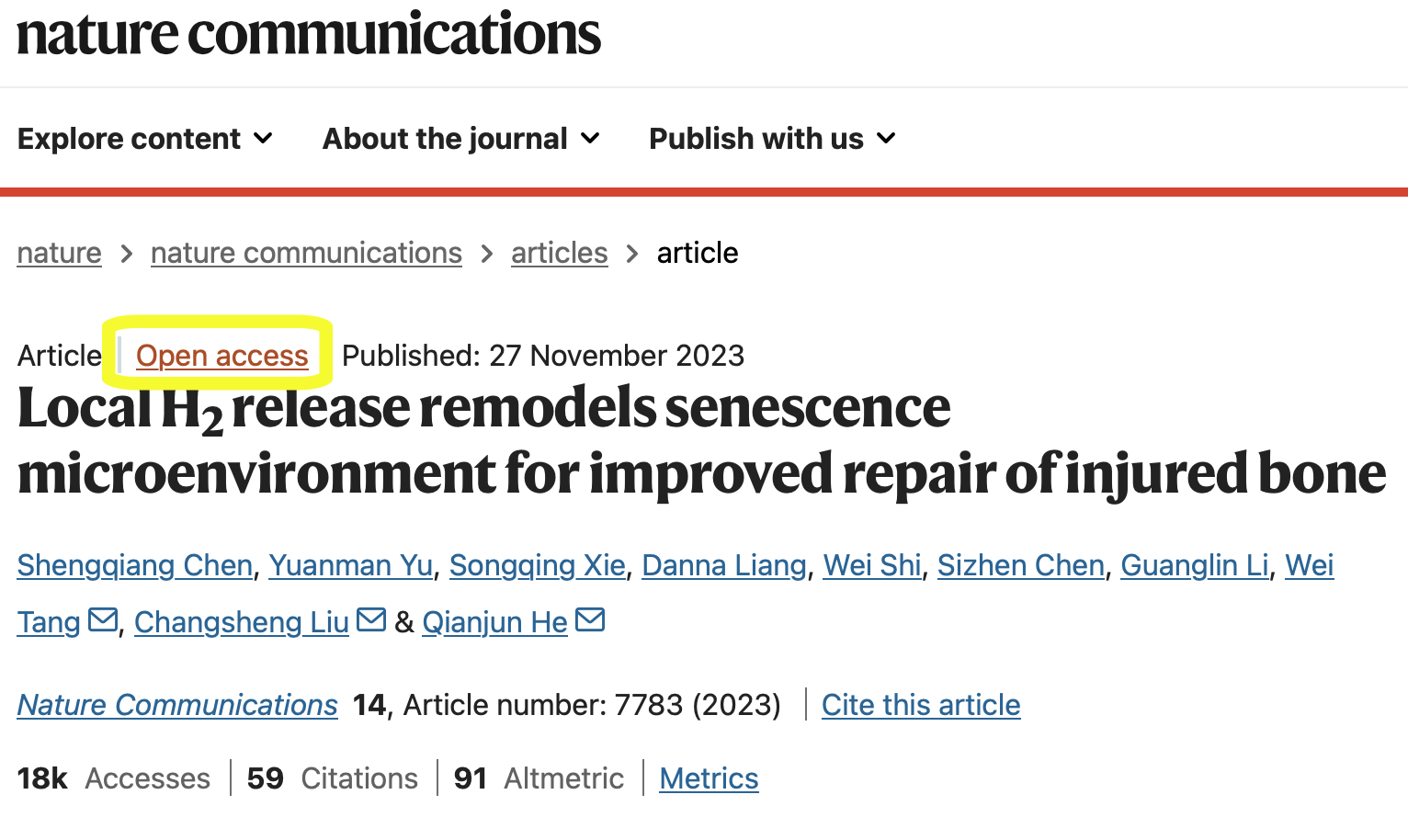


**HydroWrap for T2DM-Related Fractures: A smart H2S-delivery controller modulating Macrophage senescence**


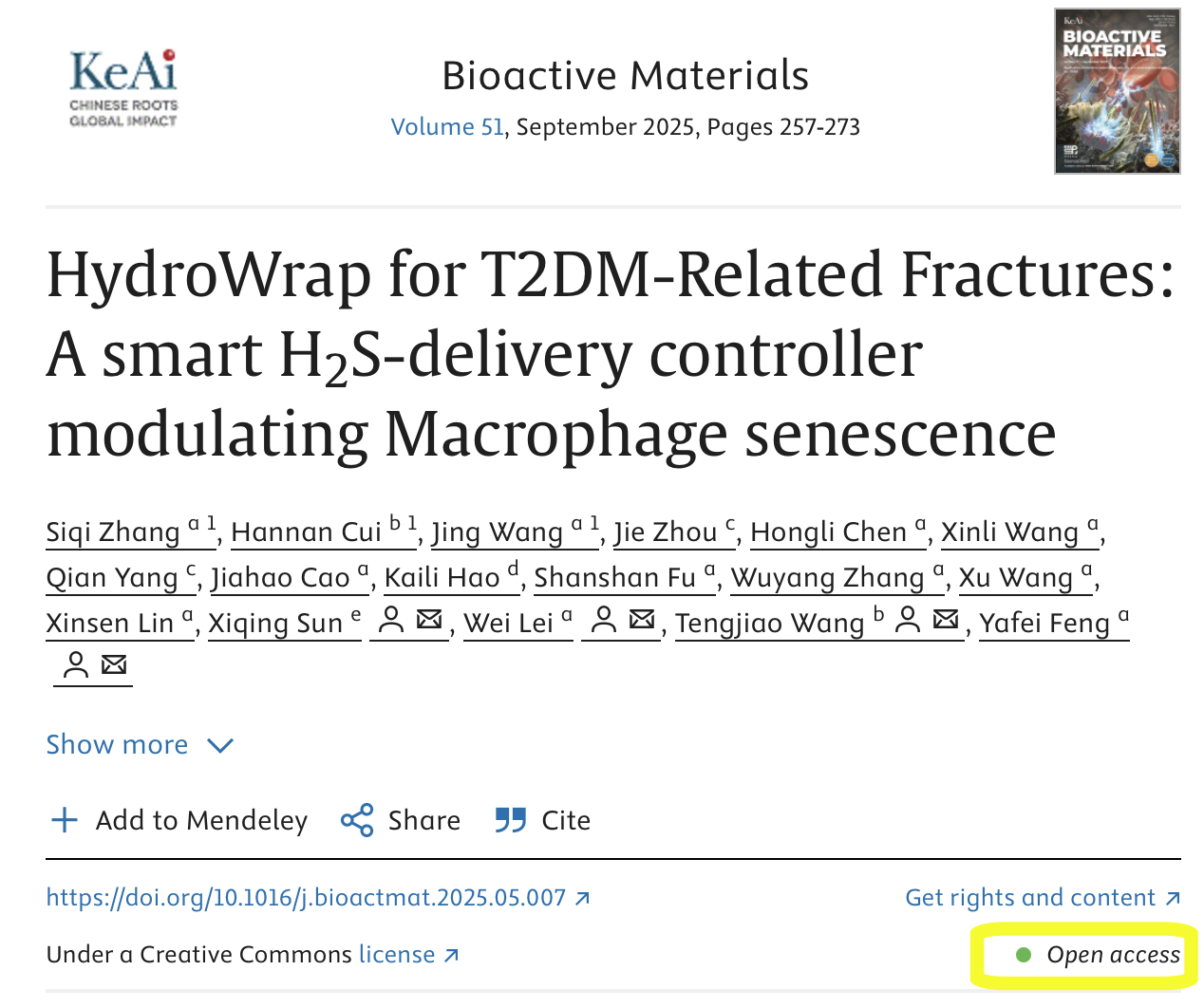


**Attenuation of osteoarthritis progression via locoregional delivery of Klotho-expressing plasmid DNA and Tanshinon IIA through a stem cell-homing hydrogel**


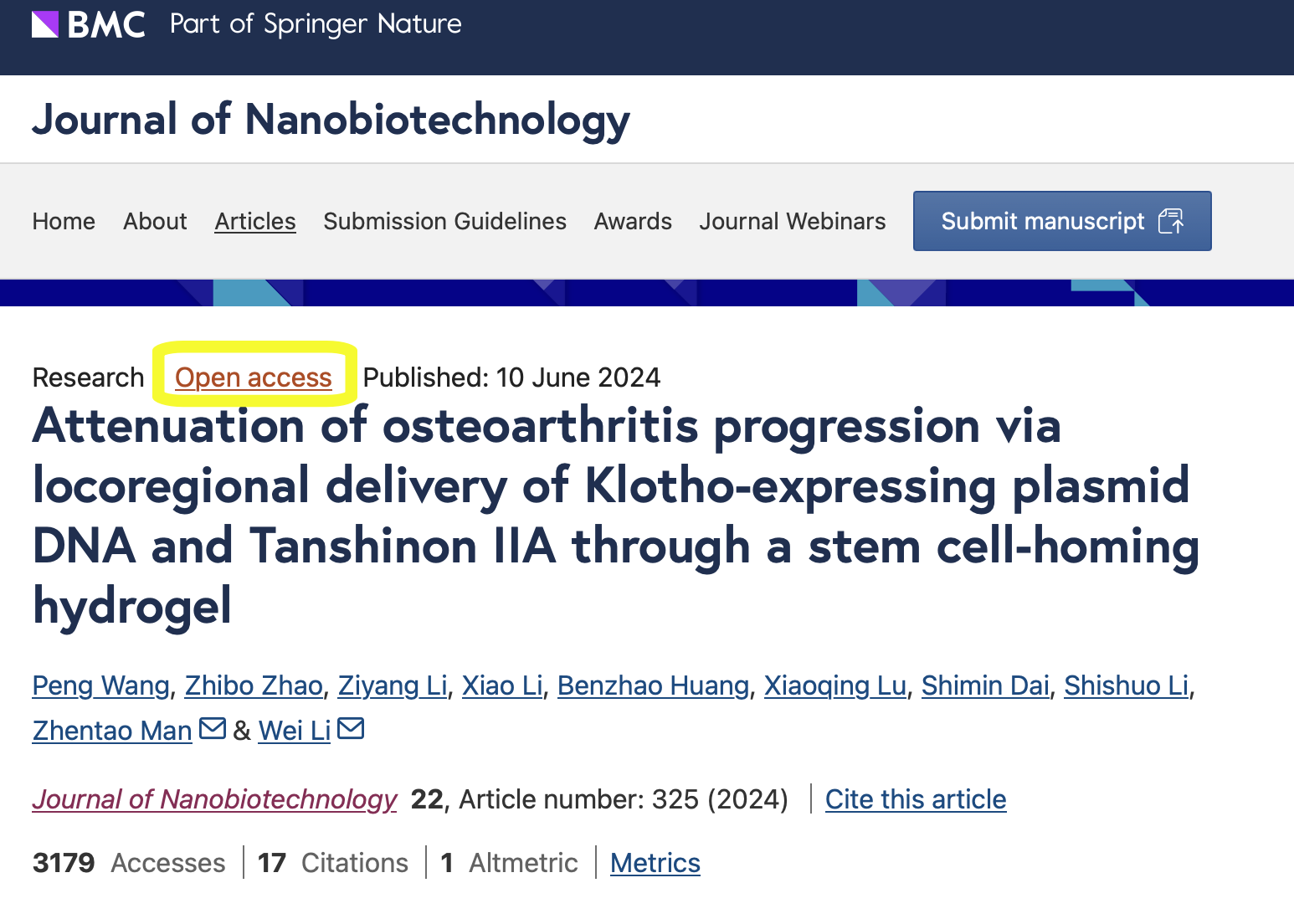

Supplement: Multimedia component 1 [file mmc1.docx]
